# Effects of *hap2* deletion on *mnp*/*vp* transcription in *Pleurotus ostreatus* grown on lignocellulosic substrates

**DOI:** 10.1007/s00253-024-13352-7

**Published:** 2024-11-13

**Authors:** Keita Kayama, Takehito Nakazawa, Iori Yamaguchi, Moriyuki Kawauchi, Masahiro Sakamoto, Yoichi Honda

**Affiliations:** Graduate School of Agriculture, Kyoto University, Sakyo-ku, Kyoto, 606-8502 Japan

**Keywords:** Basidiomycete, Mushroom, CCAAT, Gene regulation, Lignin, White-rot fungi

## Abstract

**Abstract:**

The regulatory mechanisms governing expression of genes encoding lignin-modifying enzymes (LME) in white-rot fungi remain largely unexplored. Although molecular cloning has identified CCAAT-boxes frequently located 5′-upstream of these genes, their role in transcriptional regulation is not well understood. This study examines the function of *hap2*, a gene encoding a hypothetical protein homologous to a component of the CCAAT-binding Hap complex, in the white-rot fungus *Pleurotus ostreatus*. Deletion of *hap2* resulted in significantly reduced Mn^2+^-dependent peroxidase activity and lignin-degrading capacity compared to the parental strain 20b grown on beech wood sawdust (BWS) medium. Real-time PCR revealed that *vp2* transcript levels were significantly lower in *hap2* deletants than in 20b grown when cultured on the three solid media consisting of BWS, holocellulose, or Avicel, but not on yeast-malt-glucose (YMG) agar plates. Additionally, glutathione *S*-transferase (GST) pulldown and electrophoretic mobility shift assays demonstrated that recombinant *P*. *ostreatus* Hap2, Hap3, and Hap5 expressed in *Escherichia coli* form a complex capable of binding to the CCAAT sequence 5′-upstream of *vp2* in vitro. These results suggest that Hap2, as part of the CCAAT-binding complex, is essential for transcriptional upregulation of *vp2* in *P*. *ostreatus* growing on lignocellulosic substrates.

**Key points:**

• *P. ostreatus hap2 deletants were generated.*

• *Lignin-degrading capacity was significantly reduced in the hap2 deletants.*

• *vp2 was significantly downregulated upon hap2 deletion.*

**Supplementary Information:**

The online version contains supplementary material available at 10.1007/s00253-024-13352-7.

## Introduction

White-rot fungi, classified under the class *Agaricomycetes*, are integral to the global carbon cycle due to their exceptional ability to decompose lignocellulose. This complex material, primarily composed of cellulose, hemicellulose, and lignin, is notoriously resistant to degradation (Suryadi et al. 2022), yet white-rot fungi excel in breaking it down through the secretion of specialized enzymes, such as lignin peroxidase (LiPs), manganese peroxidases (MnP), and versatile peroxidases (VPs). These lignin-modifying enzymes (LMEs) are essential for lignin degradation (Martínez 2002; Nakazawa et al. 2023b). The expression levels of genes encoding LMEs vary depending on culture conditions and time periods, as observed in several white-rot fungi, such as *Pleurotus ostreatus* (Fernández-Fueyo et al. 2014; Alfaro et al. 2016; Yoav et al. 2018), *Phanerochaete chrysosporium*, and *Gelatoporia subvermispora* (Hori et al. 2011, 2014; Fernandez-Fueyo et al. 2012)*.* This variation likely allows the fungi to efficiently decompose wood biomass under specific environmental conditions. Generally, the expression levels of LME-encoding genes are higher when fungi are grown on lignocellulosic substrates when compared to non-lignocellulosic media (Hori et al. 2014; Fernández-Fueyo et al. 2016). Understanding the regulatory mechanisms controlling expression of LME genes is vital for development of biotechnological applications, such as eco-friendly lignin removal from lignocellulose to enhance saccharification and conversion into valuable chemical products.

Transcriptional expression in response to environmental factors, such as nutrient availability, occurs not only in white-rot fungi, but also in many other fungal species (Mach et al. 1996; Gielkens et al. 1999; Tsukagoshi et al. 2001). Transcription typically begins when RNA polymerase binds to the promoter region near the transcription start site (TSS), facilitated by general transcription factors. Typical eukaryotic RNA polymerase II core promoters contain sequences like TATA-boxes (Basehoar et al. 2004), which are required for transcription and are usually located 20–40 bp upstream of the TSS (Bucher 1990). The TATA-binding protein functions as a subunit of the TFIID coactivator complex (Lemon and Tjian 2000; Bhaumik and Green 2002). In addition, *cis*-regulatory elements where transcriptional repressor/activators bind to regulate transcription levels are typically located 5′-upstream of the core promoter region. The CCAAT-box, typically found 50–200 bp upstream of the TSS (Bucher 1990), is a well-studied element in eukaryotes, including fungal species such as *Aspergillus nidulans* and *Cryptococcus neoformans* (Steidl et al. 1999; Kim and Bahn 2022). The CCAAT-binding Hap heterotrimeric complex (nuclear factor Y in mammals) is highly conserved across eukaryotes (Nardone et al. 2017). Other transcription factors, such as the CCAAT/enhancer binding protein (Ramji and Foka 2002), can also bind to CCAAT sequences; however, they primarily recognize palindromic sequences rather than CCAAT specifically (Kato 2005).

Molecular cloning studies have previously identified CCAAT and TATA boxes in the 5′-upstream regions of genes encoding LiP and MnP (Schalch et al. 1989; Camarero et al. 2000). Nguyen et al. (2020) demonstrated that deletion of the TATA sequence in the *P*. *ostreatus mnp3* promoter significantly impaired basal transcriptional expression. However, the roles of CCAAT boxes which regulate the transcription of LME-encoding genes remains unexplored. To advance our understanding of the transcriptional regulation of these genes, we investigated that effects of deleting the *hap2* gene encoding for a protein homologous to a component of the CCAAT-binding complex, on *mnp*/*vp* transcription in *P*. *ostreatus*. In addition, we analyzed the transcriptional expression patterns of *mnp* and *vp* genes on different solid media, primarily composed of lignocellulose or its component(s).

## Materials and methods

### Strains, media, and culture conditions

The *P*. *ostreatus* strains used in this study are listed in Table 1. Yeast and malt extracts with glucose (YMG) medium (Rao and Niederpruem 1969) solidified with 2% (w/v) agar in 9 cm Petri dishes were used for routine cultures.

**Table 1:** The *P*. *ostreatus* strains used in this study

Strain	Genotype/description	Source
20b	*A2B1 ku80*::*cbx*^*R*a^ / a *ku80* deletant from PC9^b^	Salame et al. (2012b)
Δ*hap2*#1	*A2B1 ku80*::*cbx*^*R*a^ *hap2*::*hph* / a *hap2* deletant derived from 20b	This study
Δ*hap2*#2	*A2B1 ku80*::*cbx*^*R*a^ *hap2*::*hph* / a *hap2* deletant derived from 20b	This study

^a^*cbx*^*R*^ indicates the carboxin resistance gene (Honda et al. 2000)

^b^Spanish type culture collection accession number CECT20311 (Larraya et al. 1999)

We used three different lignocellulose-based media: beech (*Fagus crenata*) wood sawdust medium (extracted BWS-I used for RNA and extracellular enzyme experiments; non-extracted BWS-II for analysing lignin degradation), Avicel-I (crystalline cellulose) medium, and Holocellulose-I medium in this study. The compositions of these lignocellulosic media are listed in Supplemental Table S1. Wheat bran was added to these media to enhance fungal growth and production of lignin-modifying enzymes (Pickard et al. 1999; Tsukihara et al. 2006). Beech wood sawdust, wheat bran, and Avicel PH-101 were purchased from Shinkoen (Gifu, Japan), Nisshin Seifun (Tokyo, Japan), and Sigma-Aldrich (St. Louis, MO, USA). Holocellulose was prepared from the extracted beech wood sawdust using the Jayme-Wise method (Green 1963) as described by Nakazawa et al. (2023a). Each lignocellulosic medium was prepared in 6-cm glass Petri dishes. One piece of YMG agar (approximately 1 × 1 × 1 cm) growing each *P*. *ostreatus* strain (culture period of 5–7 days) was inoculated into the center of each dish. The cultures were maintained at 28°C under continuous darkness.

*P*. *ostreatus* was transformed using protoplasts prepared from mycelial cells, as described by Salame et al. (2012b).

### Construction of plasmids and transformation of *P. ostreatus*

The plasmid for *hap2* disruption was constructed as described by Nakazawa and Honda (2015). Briefly, genomic fragments (scaffold_5:1,660,403–1,663,429 in the NCBI genome database; https://www.ncbi.nlm.nih.gov/datasets/genome/GCF_014466165.1/) containing *hap2* amplified by PCR using the genomic DNA from 20b and the primer pair TN991/TN994 (Supplemental Table S2) were cloned into pBluescript II SK + (Agilent Technologies, Santa Clara, CA, USA) digested with *Eco*RV, followed by inverse PCR using the primer pair TN992/TN993. The hygromycin resistance gene, *hph*, present in plasmid pTN24-1 (Nakazawa et al. 2017a) was amplified by PCR using the primer pair TN400/M13R and fused with the inverse PCR product using the NEBuilder Hi-Fi DNA Assembly Cloning Kit (New England Biolabs, Ipswich, MA, USA). The 5′*hap2*-P_*β-tubulin*_-*hph*-T_*β-tubulin*_-3′*hap2* cassette in the resulting plasmid was amplified using TN991/994, followed by introduction into strain 20b. Hygromycin resistance transformation was performed using protoplasts prepared from mycelial cells, as described by Salame et al. (2012a) with some modifications (Nakazawa et al. 2016b).

### Assay for extracellular enzyme activities

Each *P*. *ostreatus* strain was grown for 13 days and 20 days on BWS-I, followed by an assay for extracellular Mn^2+^- and H_2_O_2_-dependent phenol oxidase (MnP) activity using 2-methoxyphenol (Wako, Tokyo, Japan) as the substrate, as described by Kamitsuji et al. (2004) and Nakazawa et al. (2017b). The MnP activity was calculated by subtracting the activity determined in the presence of H_2_O_2_ from that in the presence of MnSO_4_ and H_2_O_2_.

The extracellular polysaccharide-hydrolyzing activities were estimated using 4-nitrophenyl (4-NP) l-arabinofuranoside (Sigma-Aldrich), 4-NP β-d-cellobioside (Tokyo Chemical Industry, Tokyo, Japan), and 4-NP β-d-xylopyranoside (Tokyo Chemical Industry) as substrates as described in Nakazawa et al. (2023a). Total cellulase and xylanase activities were also assessed using AZCL-HE-cellulose and Xylazyme AX (Megazyme, Wicklow, Ireland), respectively, as described by Nakazawa et al. (2023a).

### Quantification of Klason lignin

Each *P*. *ostreatus* strain was grown on BWS-II for 20 and 30 days, followed by quantification of the residual amount of Klason lignin (acid-insoluble) as described by Ritter et al. (1932) and Nakazawa et al. (2019).

### Quantitative reverse-transcription PCR with real-time PCR or digital PCR

Each *P*. *ostreatus* strain was grown on BWS-I, Holocellulose-I, Avicel-I (for 13 days or 20 days), and YMG agar plates overlaid with cellophane (for 7 or 16 days). Total RNA was isolated from each strain grown on BWS-I, Avicel-I, and Holocellulose-I media using a FastGene RNA Premium Kit (Nihon Genetics, Tokyo, Japan). Total RNAs was isolated from each strain grown on YMG agar plates using ISOGEN II (Nippon Gene, Tokyo, Japan), as described by Nakazawa et al. (2016a).

Quantitative reverse-transcription PCR (qRT-PCR) using the droplet digital PCR system QX200 (Biorad, Hercules, CA, USA) and the real-time PCR system QuantStudio 5 (Thermo Fisher Scientific) were performed as described by Nakazawa et al. (2023b). The primers and probes used for the digital PCR are listed in Supplemental Tables S3 and S4, respectively. The primers used for real-time PCR are listed in Supplemental Table S3.

### Glutathione S-transferase pulldown assay

cDNA fragments encoding the core region of Hap2 (amino acids 93–173) and the plasmid pGEX-6P-1 (Cytiva, Marlborough, MA, USA) were amplified by PCR using the primer pairs TN1097/TN1098 and TN744/TN746 (Supplemental Table S2), respectively. The fragments were then fused using the NEBuilder HiDi DNA Assembly Cloning Kit (New England Biolabs). The resulting plasmid was designated as pGEX-Hap2-core. pGEX-Hap2-core and pGEX-6P-1 were independently introduced into *Escherichia coli* BL21 (Takara, Shiga, Japan) to produce the fusion (bait) protein glutathione *S*-transferase (GST)-Hap2 (93–173 aa) and GST (control), respectively.

The codon-optimized fragment encoding full-length Hap3 (amino acids 1–159; amplified using TN1528/TN1529) and the cDNA fragment encoding full-length Hap5 (amino acids 1–187; amplified using TN1531/TN1532) were separately fused with the expression vectors pEFa and pEFk (FASMAC, Kanagawa, Japan), which were amplified using TN1523/TN1526 using the in-fusion HD cloning kit (Takara). The resulting constructs were designated as pEFa-Hap3 and pEFk-Hap5. These plasmids were independently transformed or co-transformed into *E*. *coli* BL21 (DE3) cells (Nippon Gene), producing Hap3, Hap5, or both Hap3 and Hap5 as prey proteins.

*E*. *coli* cells [BL21 or BL21 (DE3)] producing GST-fused bait protein, GST control protein, and prey proteins were cultured in 100 ml of 2 × YT liquid medium [polypeptone, 1.6% (w/v) g; yeast extract, 1% (w/v); NaCl, 0.5% (w/v)]. The BL21 strains producing bait protein and control protein were incubated at 28°C. IPTG was added at a final concentration of 1 mM when the OD_600_ reached 0.5, and the cells were incubated for an additional 5 h. The BL21(DE3) strain producing prey proteins were cultured at 25°C. Isopropyl β-d-1-thiogalactopyranoside (IPTG) was added to a final concentration of 0.5 mM when OD_600_ reached 0.5 and incubated for additional 5 h. Cells were harvested, washed in cold phosphate buffered saline (PBS) (137 mM NaCl, 2.7 mM KCl, 10 mM Na_2_HPO_4_, and 1.8 mM KH₂PO₄) resuspended in 10 ml of cold pulldown buffer [50 mM NaCl, 50 mM HEPES–KOH (pH 7.9), 5% (w/v) glycerol, 0.1% (w/v) Tween 20, and 1 mM phenylmethylsulfonyl fluoride (PMSF) (Nacalai Tesque, Kyoto, Japan)], and disrupted by sonication using BIORUPTOR 2 (Sonicbio, Kanagawa, Japan). The cell lysates were centrifuged at 9,500 g at 4°C for 10 min, and the resulting supernatants were stored at – 80°C until use.

For the pulldown assay, 1 ml aliquots of supernatant containing GST-Hap2 (93–173 aa) as bait or GST as control were mixed with 10 µl Glutathione High Capacity Magnetic Agarose Beads (Sigma-Aldrich) at 4°C for 40 min. The beads were collected, washed once with cold pull-down buffer, and mixed with 1 ml of the supernatant containing Hap3, Hap5, or both Hap3 and Hap5. After incubating the mixtures at 4°C for 40 min, the beads were washed three times and boiled in 50 µl 2 × SDS sample buffer [125 mM Tris–HCl (pH 6.8), 10% (v/v) 2-mercaptoethanol, 4% (w/v) sodium dodecyl sulfate (SDS), 10% (w/v) sucrose, and 0.01% (w/v) bromophenol blue]. Samples were separated using 15% SDS–polyacrylamide gel and stained with CBB Stain One Super (Nacalai Tesque, Kyoto, Japan).

### Electrophoretic mobility shift assay

Single-stranded oligonucleotide TNS12, which includes the sequence 5′-upstream of the *vp2* gene start codon from − 69 bp to − 89 bp and contains a CCAAT sequence, and single-stranded oligonucleotide TNS13, with a mutated CCAAT sequence, were separately annealed to the Cy5-labeled oligonucleotide selex_fm2 (Supplemental Table S2). The annealed oligonucleotides were extended using Klenow fragment (Takara) to produce 5 µM double-stranded fluorescent probes. The probe derived from TNS12 containing the CCAAT sequence was designated as probe 1, whereas that derived from TNS13 containing the mutated sequence was designated as probe 2.

A non-specific competitor was prepared by annealing single-stranded Selex_R with single-stranded Selex_Rdm_comp with a random 20-bp sequence, followed by extension using Klenow. Specific competitors were prepared from KYK136 and KYK137 (Supplemental Table S2).

The plasmid pEFa-GST-Hap3 containing codon-optimized GST-Hap3 (amino acids 1–159) was synthesized using FASMAC (Kanagawa, Japan). The cDNA fragment encoding full-length Hap5 (aa 1–187; amplified using TN1530/TN1532) was fused with the fragment of pEFa-GST-Hap3, excluding the coding region of Hap3, which was amplified using TN1524/TN1526 using the in-fusion HD cloning kit (Takara). The resulting plasmid was designated pEFa-GST-Hap5. pGEX-Hap2-core was introduced into *E*. *coli* BL21, whereas pEFa-GST-Hap3 and pEFa-GST-Hap5 were independently introduced into *E*. *coli* BL21 (DE3). The process from each *E*. *coli* culture to supernatant storage was carried out as described in the previous section on the GST pulldown. One milliliter aliquots of supernatant containing GST-Hap2 (93–173 aa), GST-Hap3, and GST-Hap5 were mixed with 200 µl Glutathione High Capacity Magnetic Agarose Beads (Sigma-Aldrich) at 4°C for 40 min. The beads were collected, washed thrice with cold pull-down buffer, and eluted with elution buffer [50 mM Tris–HCl (pH 8.1) and 50 mM glutathione].

Electrophoretic mobility shift assay (EMSA) was performed using the prepared probes, protein samples, and their competitors. The 5 × binding buffer was prepared as previously described by Papagiannopoulos et al. (1996). The 20 µl binding reaction mixture contained 13 pmol or 2 pmol of each protein sample, 1 µg poly(dI-dC), 100 nmol dithiothreitol (DTT), 500 or 250 pmol of double strand random oligonucleotide, and 2.5 pmol fluorescent probe. First, the protein samples were mixed and incubated at room temperature for 5 min. Then, reagents other than the probe were added and incubated again for 5 min. Finally, probe 1 or 2 was added and incubated at room temperature for 30 min before being applied to the gel. As shown in Fig. 8, a specific competitor with a 1600-fold molar excess relative to the probe was added before the probe. Samples were run on a 5% acrylamide gel in 0.5 × TBE buffer. The gel was pre-run at 100 V for 1 h at 4°C before loading the binding samples. Electrophoresis was performed at 150 V for 45 min at 4°C. Cy5 fluorescence was detected using an Amersham Imager 680 (Cytiva; excitation at 630 nm).

## Results

### Both TATAA- and CCAAT-boxes were detected in the 5′-upstream of *mnp/vp/lip* genes in genomes across the genomes of three white-rot fungi

To comprehensively investigate the presence or absence of CCAAT-box (5′-CCAAT-3′ or complementary ATTGG; Zeilinger et al. 2001) and TATA-boxes (TATAA) in the LME-encoding genes, we analyzed the 5′-upstream sequences of LME-encoding genes predicted in the Joint Genome Institute (JGI) genome database for three white-rot fungi: *P*. *ostreatus* (https://mycocosm.jgi.doe.gov/PleosPC9_1/PleosPC9_1.home.html), *G. subvermispora* (https://mycocosm.jgi.doe.gov/Cersu1/Cersu1.home.html), and *P. chrysosporium* (https://mycocosm.jgi.doe.gov/Phchr2/Phchr2.home.html). The results, detailed in Table 2 and Supplemental Tables S5, and Table S6, reveal that in *P*. *ostreatus*, seven out of the total nine *mnp*/*vp* genes contain both TATAA- and CCAAT-boxes within their 5′-upstream regions, except for *mnp4* and *mnp5*. In contrast, none of the *lac* genes contained either the TATA-box or CCAAT-box. In *G*. *subvermispora*, both TATA-box and CCAAT-box are present in the 5′-upstream sequences of 10 genes out of the total 13 *mnp* genes (except for *mnp2*, *mnp9*, and *mnp12*), of one gene out of the total two *lip* genes, and of three genes out of the total seven laccase-encoding genes (*lcs1*–*7*). In *P*. *chrysosporium*, both TATAA-box and CCAAT-box are present in the 5′-upstream sequences of three genes out of the total five *mnp* genes (except for *mnp3*, and *mnp5*), and of six genes out of the total ten *lip* genes. These results suggest that CCAAT boxes are common features in the 5′-upstream sequences of many lignin-modifying peroxidase-encoding genes in white-rot fungi, potentially playing a critical role in their transcriptional regulation.

**Table 2:** Promoter analysis of *P*. *ostreatus*

Gene^a^	Protein ID^b^	TATAA-box^c,d^	CCAAT-box^c,e,f^
*vp1*	116738	63	111( −), 156( +)
*vp2*	60432	59	86( −)
*vp3*	123383	73	91( −)
*mnp1*	115087	74	152( +)
*mnp2*	61491	73	153( −)
*mnp3*	51690	62	132( −)
*mnp4*	121638	84	–
*mnp5*	52120	–	101( −), 263( −)
*mnp6*	51713	88	167( +), 198( −)
*lac1*	90578	–	146( −)
*lac2*	116143	–	206( +)
*lac3*	123288	–	89( −)
*lac4*	65894	–	238( −)
*lac6*	81104	57	–
*lac7*	60400	57	–
*lac9*	81107	–	175( +)
*lac10*	81117	–	–
*lac11*	90573	51	–
*lac12*	90834	51	–

^a,^^b^Gene names and protein IDs are based on the genome database of *P. ostreatus* PC9 (https://mycocosm.jgi.doe.gov/PleosPC9_1/PleosPC9_1.home.html)

^c^The distances of the TATAA-box and CCAAT-box from the start codon are shown

^d^Only TATAA-boxes located within 100 bp from the start codon are shown

^e^CCAAT-boxes located within 300 bp from the start codon are shown

^f^( +) indicates the presence of CCAAT on the coding strand, while ( −) on the template strand

### Identification of *P. ostreatus* Hap2, Hap3, and Hap5

Considering that the CCAAT or ATTGG sequence was found in the 5′-upstream sequences of most of the *P*. *ostreatus mnp*/*vp* genes, we hypothesized that the CCAAT-boxes are involved in regulation of *mnp*/*vp* transcription in *P*. *ostreatus*. The Hap2/Hap3/Hap5 (HapB/HapC/HapE) trimeric complex has been previously reported as a CCAAT-binding complex in various fungi such as *Saccharomyces cerevisiae* (McNabb et al. 1995), *A*. *nidulans* (Steidl et al. 1999), and *C*. *neoformans* (Kim and Bahn 2022). However, the HAP complex has not previously been characterized in *Agaricomycetes*, including *P*. *ostreatus*. To investigate whether trimeric HAP complex is also functional in *P*. *ostreatus*, we performed a protein BLAST (BLASTp) analysis using in the NCBI refseq_protein database and *A*. *nidulans* HapB, HapC, and HapE proteins (accession nos. XP_050467982, XP_661638, and XP_664096, respectively) as query sequences to identify homologous proteins.

*P*. *ostreatus* protein (accession no. XP_036630745 in the NCBI refseq_protein database), exhibited the highest sequence identity with the *A. nidulans* HapB (Query cover: 26%, Percent Identity: 62.63%, E-value: 3.00 × 10^−28^). Reciprocal BLASTp was also performed against the amino acid sequence XP_036630745 to confirm its correspondence with the *A*. *nidulans* HapB ortholog(s). *A*. *nidulans* HapB exhibited the highest amino acid identity with that of the *P*. *ostreatus* HapB (XP_036630745) (Query cover: 22%, Percent Identity: 64.21%, *E* value: 3.00 × 10^−28^). Furthermore, the region spanning amino acids 101–156 of XP_036630745 includes pfam02045 domain [CCAAT-binding transcription factor (CBF-B/NF-YA) subunit B] in the NCBI database (https://www.ncbi.nlm.nih.gov/gene/59377414/). In other eukaryotes including *A*. *nidulans*, mammalian species, and *Arabidopsis thaliana*, this core domain consists of two α-helices: one responsible for recognizing CCAAT sequences and the other for physically interacting with Hap3 and Hap5 (Nardone et al. 2017). Therefore, we identified XP_036630745 as *P. ostreatus* Hap2. Similarly, as shown in Supplemental Tables S7 and S8, XP_036626823 and XP_036633731 were identified as *P. ostreaus* Hap3 and Hap5, respectively.

### MnP activity and lignin-degrading capacity were reduced in *P. ostreatushap2* deletants

**Fig. 1:**
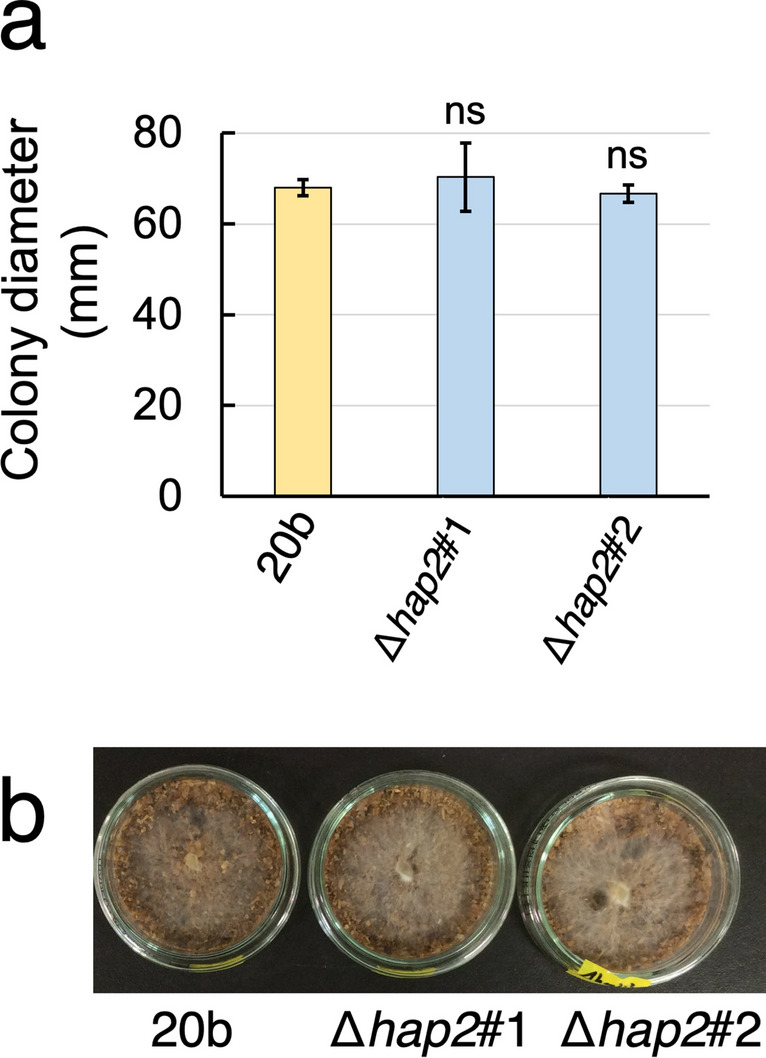
The effect of *hap2* deletion on hyphal growth rate on YMG agar plate and BWS-I. **a** Comparison of colony diameters when the indicated strains were grown on YMG. Colony diameter represents the diameter of colonies 10 days after inoculation (*n* = 3). Statistical significance tests between the indicated two strains at the respective culture periods were performed using a two-tailed equal variance *t* test. “ns” indicates statistical non-significance (*p* > 0.05). **b** An image showing hyphal growth on BWS-I at 6 d. Bar represents 3 cm

Assuming that *P*. *ostreatus* Hap2/Hap3/Hap5 functions as a CCAAT-binding complex, we investigated whether this complex is involved in the transcriptional regulation of *mnp*/*vp* in *P*. *ostreatus*. *hap2* deletants were generated from strain 20b (Table 1). The PCR-amplified *hap2*-deleting cassette was introduced into strain 20b to obtain hygromycin B-resistant transformants. As shown in Supplemental Fig. S1, genomic PCR experiments showed that two *hap2* deletants, namely Δ*hap2#*1 and Δ*hap2#*2 (Table 1), were successfully obtained. To examine whether the hyphal growth rate was affected by *hap2* deletion, 20b and the two *hap2* deletants were grown on YMG agar medium for 10 days, and colony diameters were determined. As shown in Fig. 1a, *hap2* deletants showed no significant difference in colony diameter compared to 20b. The growth rates of *hap2* deletants were similar to those of 20b grown on BWS-I (Fig. 1b). From these results, we concluded that the deletion of *hap2* does not negatively affect the hyphal growth rate under these conditions.

**Fig. 2:**
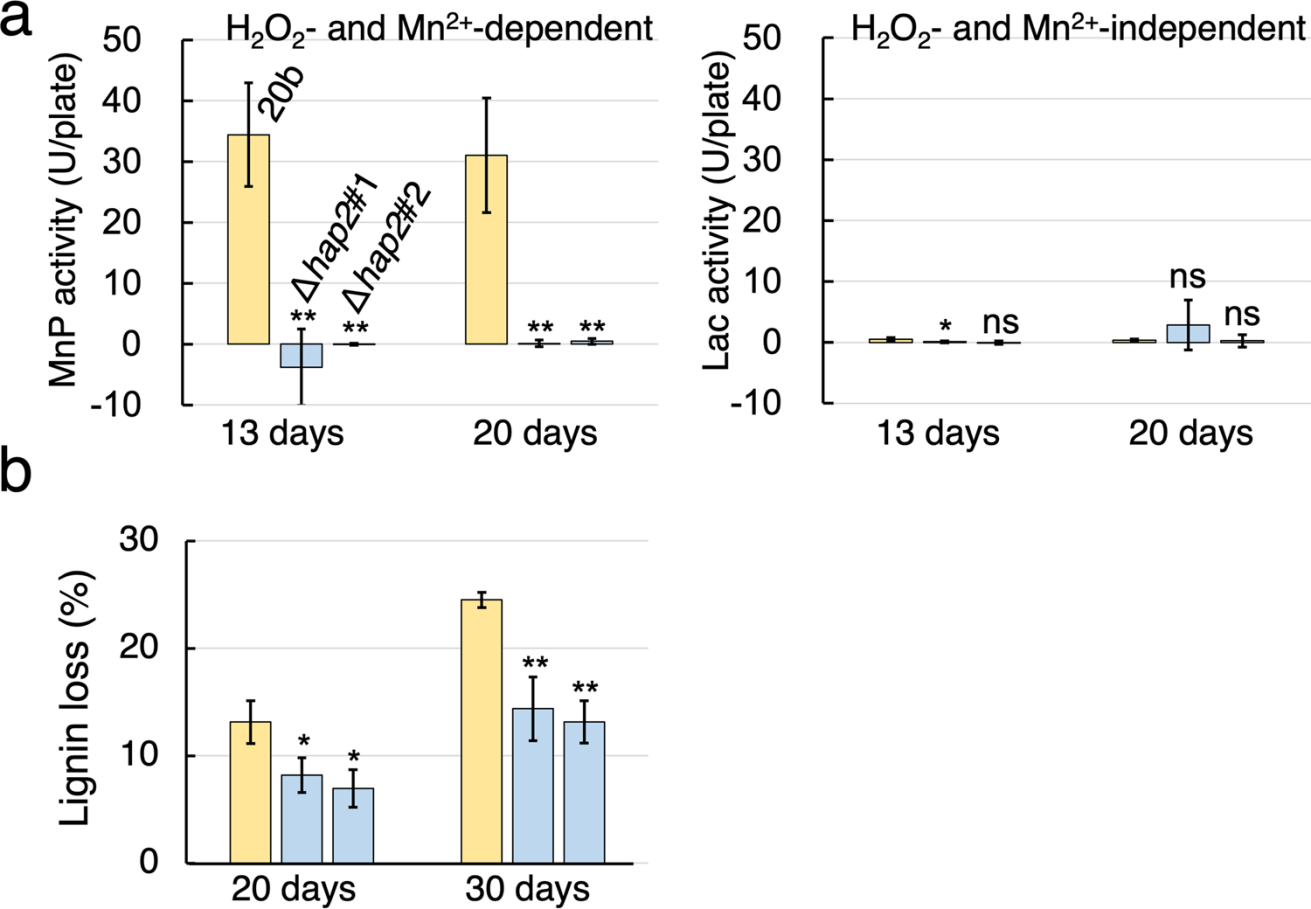
The effects of the *hap2* deletion on lignin degradation. **a** Extracellular MnP (Mn^2+^-dependent peroxidase) activity and Lac (H_2_O_2_-independent oxidase) activity of the indicated strains grown on BWS-I for 13 and 20 days (substrate is 2-methoxyphenol) (*n* = 3). One unit of activity for guaiacol oxidation was defined as the amount of enzyme that increased the absorbance at 465 nm by 1.0 per minute. **b** “Lignin loss” indicates the decreased amount of Klason lignin after growing the indicated strains on BWS-II for 20 and 30 days. The graph and bars represent the average and standard deviations, respectively. Statistical significance tests between the indicated two strains at the respective culture periods were performed using a two-tailed equal variance *t* test. *p < 0.05, **p < 0.01, ***p < 0.001, ****p < 0.0001. “ns” indicates statistical non-significance (*p* > 0.05)

To examine the effect of *hap2* deletion on expression levels of MnPs/VPs, we first evaluated extracellular MnP activity (Mn^2+^- and H_2_O_2_-dependent 2-methoxyphenol oxidizing activity) in *hap2* deletants and their parental control, 20b. Both MnPs and VPs oxidize Mn^2+^ to Mn^3+^, resulting in phenol oxidation (Gold and Glenn 1988; Ruiz-Dueñas et al. 1999). As shown in Fig. 2a, MnP activity was significantly reduced after 13 days and 20 days culturing in *hap2* deletants grown on BWS-I, whereas H_2_O_2_-independent oxidase activity was maintained. Based on this result, the decrease in the amount of Klason lignin after growing each strain on BWS-II was compared to examine whether the loss of MnP activity in *hap2* deletants reduced their lignin-degrading capacities. As shown in Fig. 2b, the decrease in the amount of Klason lignin in the media after growing *hap2* deletants was significantly lower than that after growing their parental control, 20b (Table 1). These results suggest that the production of MnPs/VPs and thus their lignin-degrading capacities are reduced in *hap2* deletants.

**Fig. 3:**
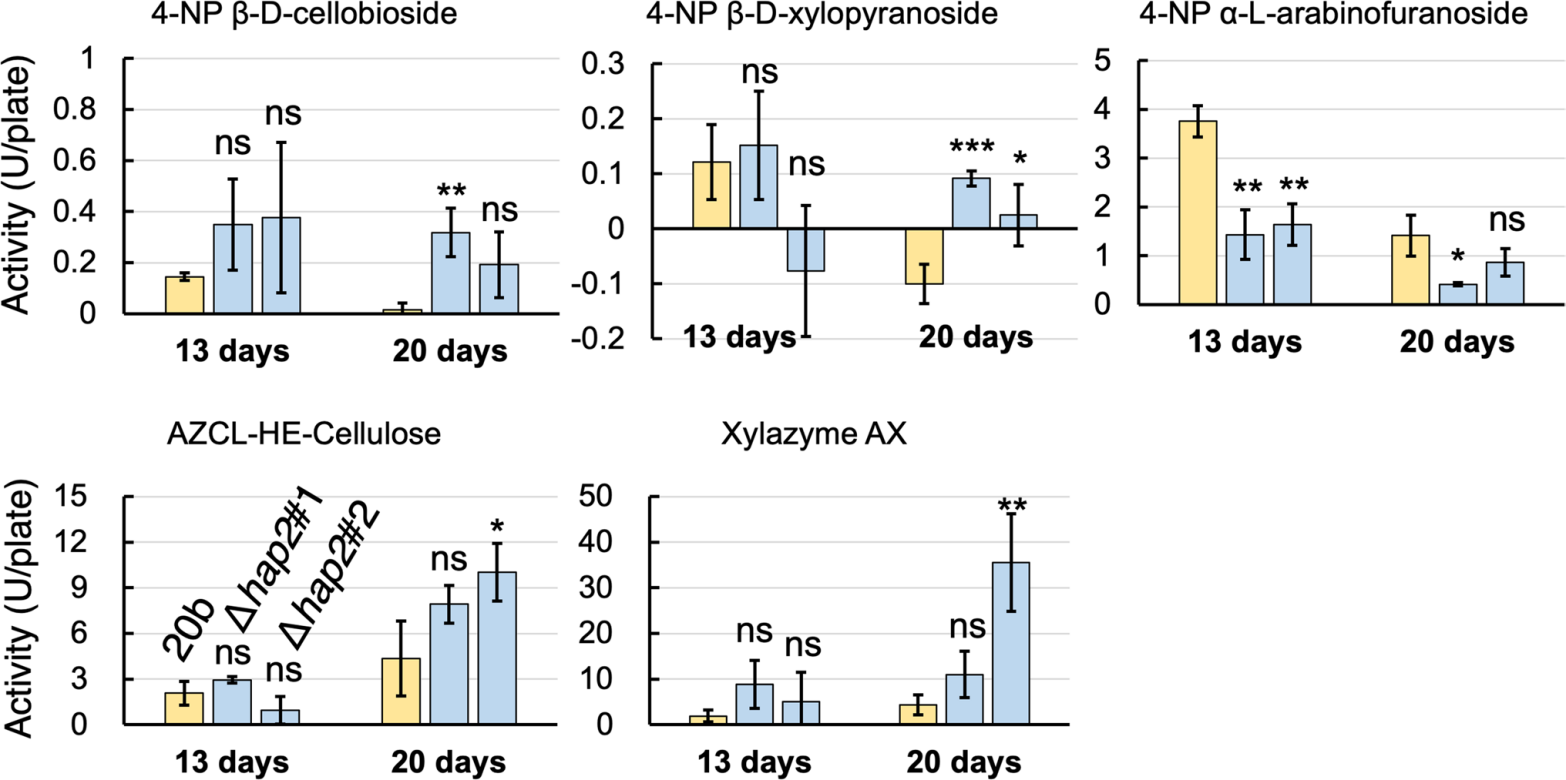
Extracellular polysaccharide-degrading activities of 20b and *hap2* deletants grown on BWS-I in the presence of the indicated substrates. Graphs and bars show the means and standard deviations, respectively (*n* = 3). One unit for the three 4-NP-coupled substrates was defined as the amount of enzyme required to release 1 μmol of 4-nitrophenol per hour. That for the remaining two substrates was defined as the amount of enzyme required to increase the absorbance at 590 nm (1-cm path length) by 1 h. Statistical significance tests between the 20b and the *hap2* deletants were performed using a two-tailed equal variance *t* test. *p < 0.05; **p < 0.01; ***p < 0.001; ****p < 0.0001. “ns” indicates statistically no significance (*p* > 0.05)

We also examined extracellular hydrolase activities using three 4-NP-coupled substrates and insoluble chromogenic substrates by using endo-cellulase and xylanase assays, AZCL-HE-Cellulose and Xylazyme AZ, respectively. As shown in Fig. 3, significantly lower activities for 4-NP α-l-arabinofuranoside were observed in the *hap2* deletants at 13 days. As extracellular enzyme activities are related to cellulose and xylan degradation, significantly higher activities for 4-NP β-l-xylopryranoside were also observed in both the *hap2* deletants at 20 days.

### *mnp/vp* transcript is abundant in wild-type strain grown on lignocellulose-based media

Based on the results shown in the previous section, we also compared the transcript abundance of *mnp*/*vp* between *hap2* deletants and 20b using real-time PCR to examine transcript abundance of these genes.

Prior to this experiment, we quantified the *mnp*/*vp* transcript copies in strain 20b grown on three types of lignocellulose-based solid media (BWS-I, Holocellulose-I-I, and Avicel-I) and YMG agar plates using droplet digital PCR. These lignocellulose-based media were used to investigate which lignocellulosic component(s) affected the transcriptional expression patterns. We also investigated *mnp*/*vp* transcript copies on YMG agar plates, as lignocellulosic components were not abundant in this medium. The copies of BWS-I have already been investigated in a previous study (Nakazawa et al. 2023b), yet with only two repeats (*n* = 2). An additional experiment was performed once more in this study, bringing the three biological replicates.

Transcriptional patterns of *mnp*/*vp* are affected by the culture period (Fernández-Fueyo et al. 2014; Alfaro et al. 2016). Therefore, we quantified the transcript copies at two culture periods for each medium: 13 days and 20 days for the three lignocellulose-based media and 7 days and 16 days for YMG. When 20b was grown on BWS-I or Avicel-I for 13 days, the mycelia covered the entire plate. On Holocellulose-I, mycelia covered only 30–50% (approximately 2–3 cm in diameter on 6-cm glass plates) at 13 days, and mycelia covered the entire plate in all lignocellulose-based media at 20 days. When grown on YMG agar medium for 7 days, mycelia covered approximately half of the plate (approximately 4.5 cm in diameter on 9-cm agar plates). At 16 days of culture, the mycelia covered the entire plate (data not shown).

**Fig. 4:**
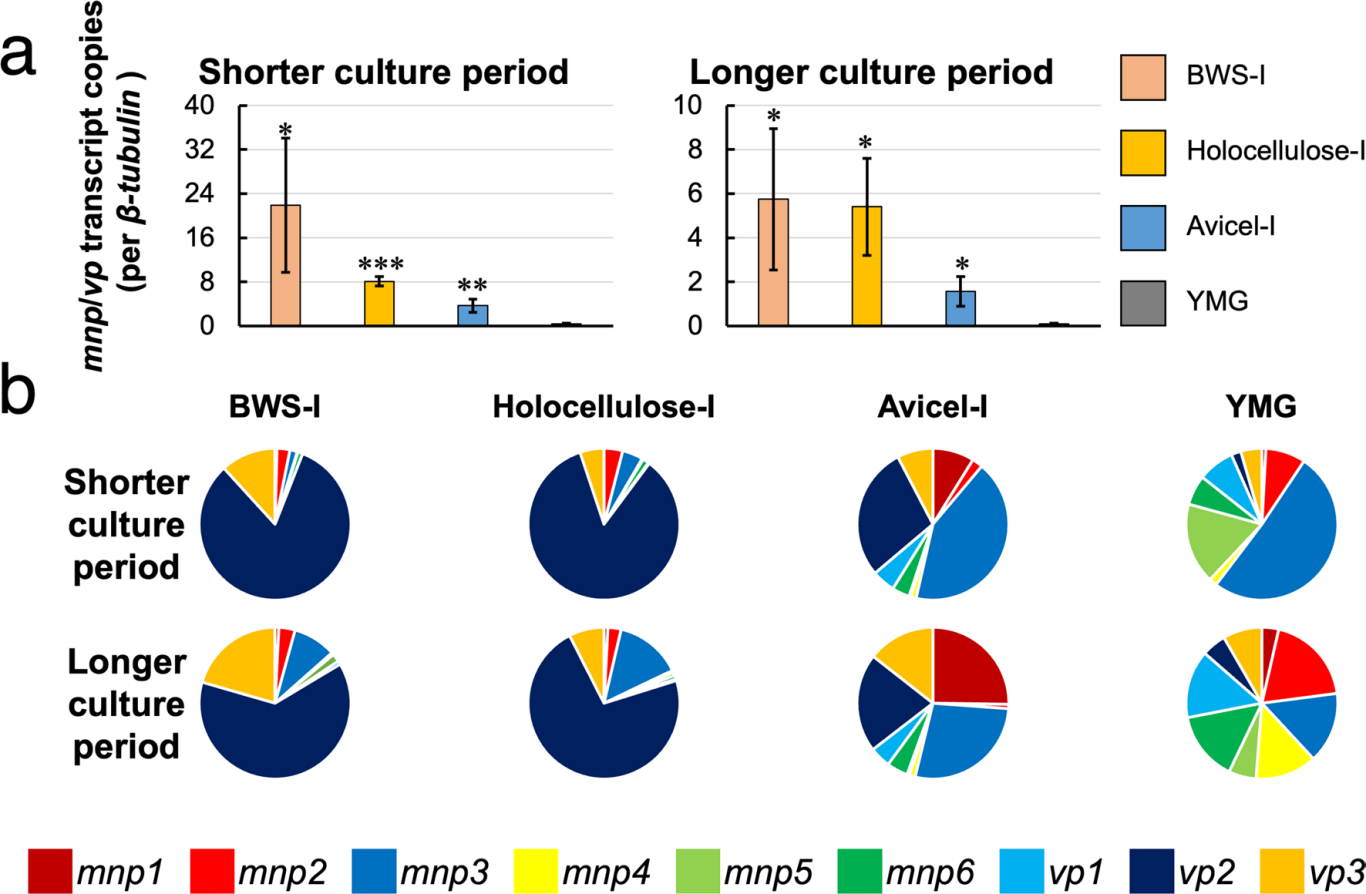
Quantification of *mnp*/*vp* transcript by digital PCR at two culture periods. **a** Sum of transcript copies of all nine *mnp*/*vp* per that of the *β*-*tubulin* (*n* = 3). “Shorter culture period” and “Longer culture period” for YMG were 7 days and 16 days, respectively. Those for the BWS-I, Holocellulose-I, and Avicel-I were 13 days and 20 days, respectively. Graphs indicate mean values and bars indicate standard deviations. Statistical significance tests between the indicated media and YMG medium were performed using a two-tailed equal variance *t* test. *p < 0.05, **p < 0.01, ***p < 0.001, ****p < 0.001. **b** Proportion of each gene in the total copies of *mnp*/*vp* (*n* = 3). The transcript copy of each gene per *β-tubulin* transcript copy was used

The total transcript copies of *mnp*/*vp* genes per transcript copy of *β-tubulin* is shown in Fig. 4a. Compared with YMG at 7 d (i.e., shorter culture period), the total transcript copies of *mnp*/*vp* were 58, 21, and 10 times higher when grown on BWS-I, Holocellulose-I, and Avicel-I for 13 days, respectively. Compared with YMG at 16 days (i.e., longer culture period), the total transcript copies of *mnp*/*vp* were 63, 60, and 20 times higher when grown on BWS-I, Holocellulose-I, and Avicel-I for 20 d, respectively. These differences were statistically significant. These results suggest that expression level of *mnp*/*vp* increases when *P*. *ostreatus* is grown on the three lignocellulosic-based media used in this study.

The percentage of transcripts from each gene among the total *mnp/vp* transcript copies is shown in Fig. 4b. In BWS-I, *vp2* accounted for approximately 60–80% of the total *mnp*/*vp* transcript copies. The transcript copy number of *vp3* was also relatively high, accounting for 10–20%. In Holocellulose-I, *vp2* accounts for approximately 70–80% of the total *mnp*/*vp* transcript copies. On Avicel-I, *vp2* and *mnp3* accounted for approximately 20–30% and 30–40% of the total *mnp*/*vp* transcript copies, respectively. These results suggest that *vp2* (VP2) was predominant in BWS-I and Holocellulose-I. In addition, the level of expression of *vp2* was on YMG agar plates was insignificant.

### Reduced *vp2* transcription in the *hap2* deletants grown on lignocellulose-based media

**Fig. 5:**
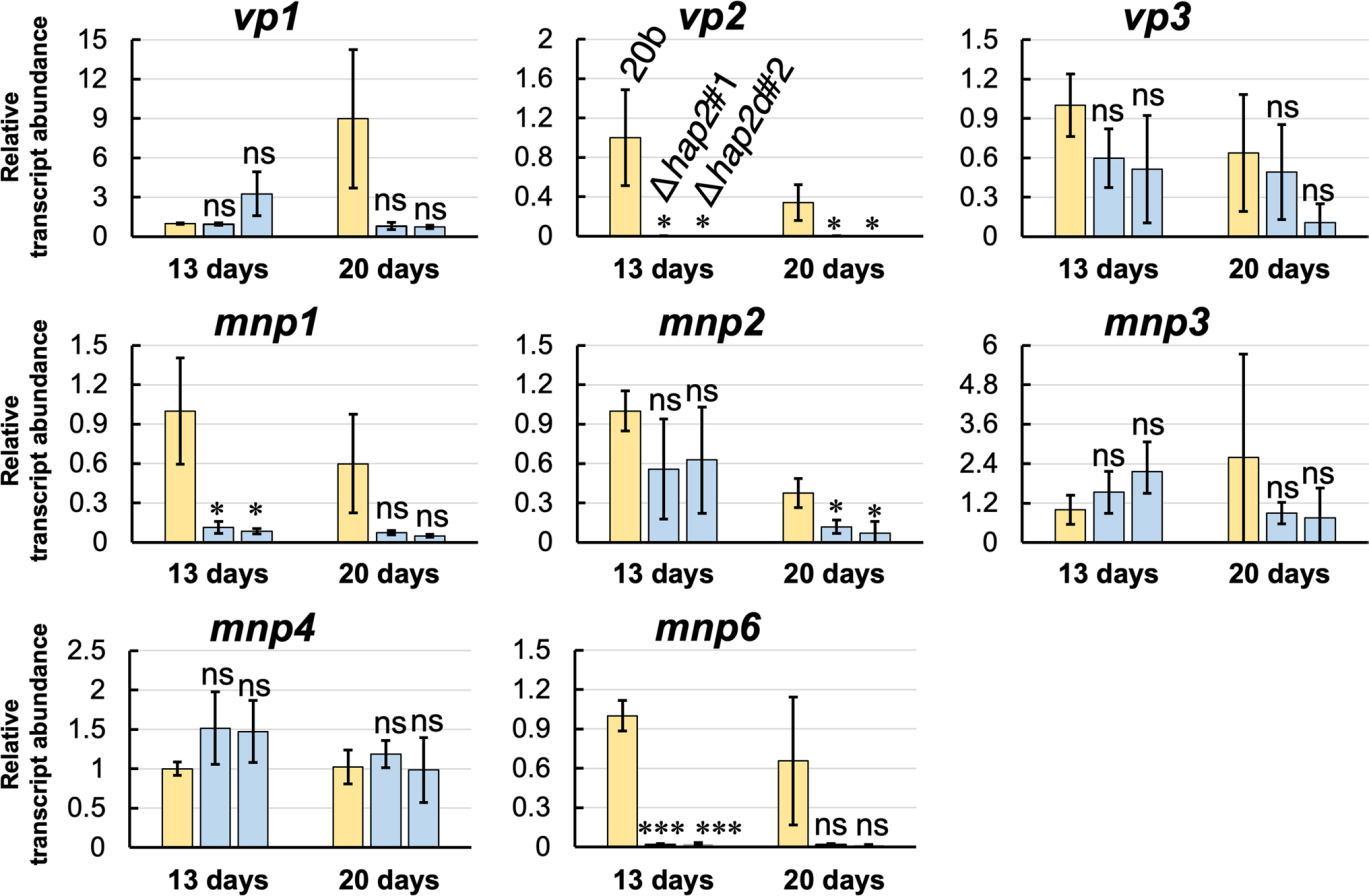
Relative transcriptional expression levels of the eight *mnp*/*vp* genes in the *hap2* deletants grown on BWS-I at 13 days and 20 days (*n* = 3). Expression levels were standardized by *β*-*tubulin*, and the expression level of 20b at 13 days is shown as 1. Graphs indicate mean values and bars indicate standard deviations. Statistical significance tests between the indicated two strains at the respective culture periods were performed using a two-tailed equal variance *t* test. *p < 0.05, **p < 0.01, ***p < 0.001, ****p < 0.0001. “ns” indicates statistical non-significance (*p* > 0.05)

The *hap2* deletants and 20b were grown on BWS-I to compare the transcript abundance of each *mnp*/*vp* using real-time PCR (Fig. 5). Although six *mnp* and three *vp* genes were predicted in the JGI genome database of *P*. *ostreatus* PC9 (Knop et al. 2015), we were unable to detect or quantify *mnp5* transcripts using previously reported primer pairs (Supplemental Table S3). This may be due to the highly suppressed transcription of *mnp5* in the PC9/20b-based strains (Fig. 4; Wu et al. 2020). Therefore, we analyzed the remaining eight *mnp*/*vp* transcripts. As shown in Fig. 5, the transcriptional abundance of *vp2* was significantly lower in *hap2* deletants than in 20b at both 13 days and 20 days. In addition, the levels of *mnp1* and *mnp6* were significantly lower in *hap2* deletants at 13 days.

**Fig. 6:**
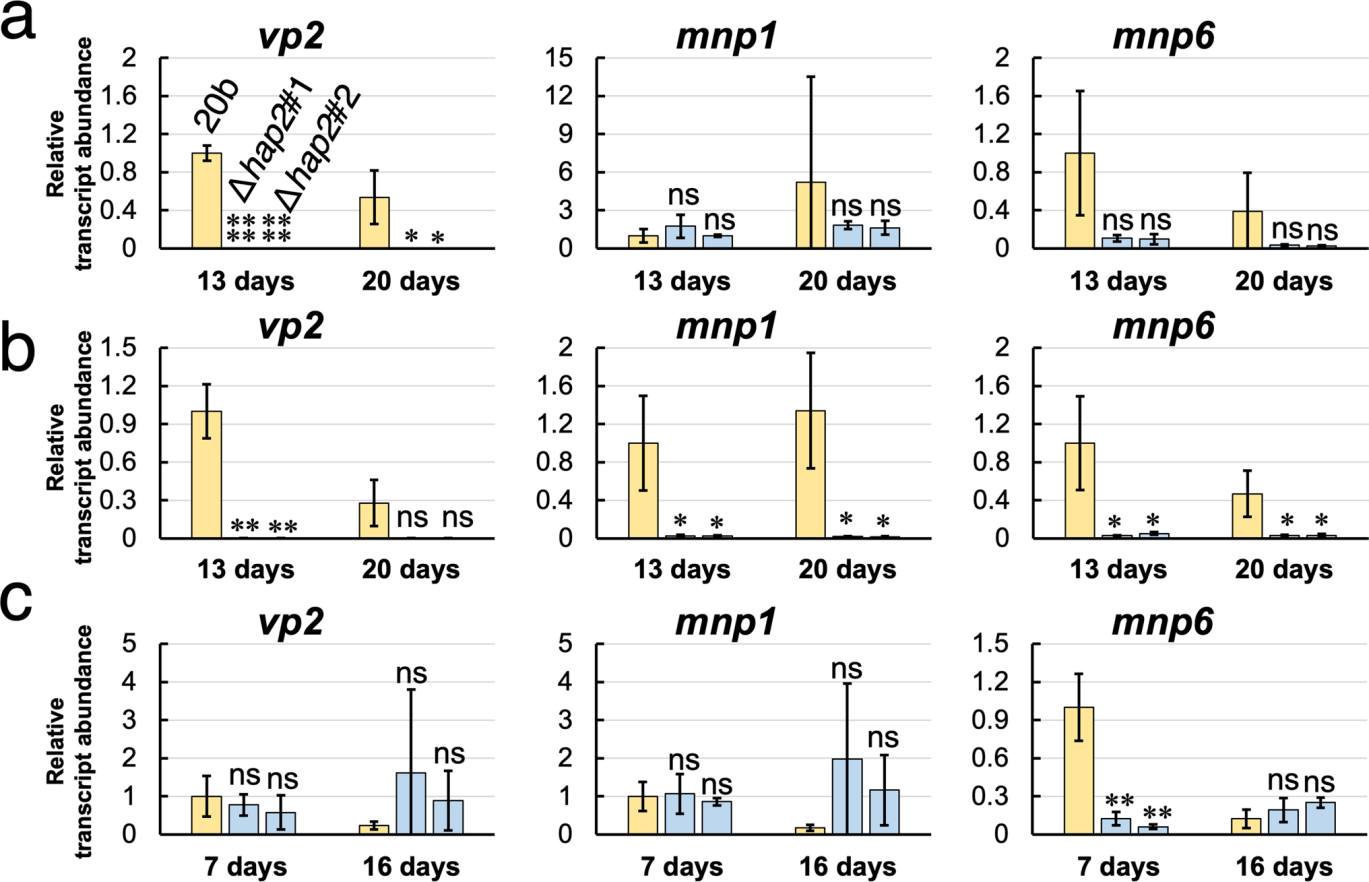
Relative transcriptional expression levels of *vp2*, *mnp1*, and *mnp6* in the *hap2* deletants grown on **a** Holocellulose-I, **b** Avicel-I, and **c** YMG agar plate at 13 days and 20 days (*n* = 3). Expression levels were standardized by *β*-*tubulin*, and the expression level of 20b at 13 days is shown as 1. Graphs indicate mean values and bars indicate standard deviations. Statistical significance tests between the indicated two strains at the respective culture periods were performed using a two-tailed equal variance *t* test. *p < 0.05, **p < 0.01, ***p < 0.001, ****p < 0.0001, *****p < 0.00001. “ns” indicates statistical non-significance (*p* > 0.05)

Next, we compared the transcript abundance of each *mnp*/*vp* when *P*. *ostreatus* strains were grown on Holocellulose-I, Avicel-I, and YMG agar plates (Supplemental Figs. S2–S4). Similar to BWS-I, significantly lower transcript abundance of *vp2*, *mnp1*, and *mnp6* observed in some cases (Fig. 6). In particular, a significantly lower transcript abundance of *vp2* was observed in *hap2* deletants grown on Holocellulose-I, Avicel-I, and BWS-I in most cases. As for transcript abundance on the YMG agar plates, that of *mnp6* at 7 d was significantly lower in *hap2* deletants (Supplemental Fig. S4).

### The recombinant *P. ostreatus* Hap multimer binds specifically to the CCAAT sequence 5′-upstream of *vp2* in vitro

**Fig. 7:**
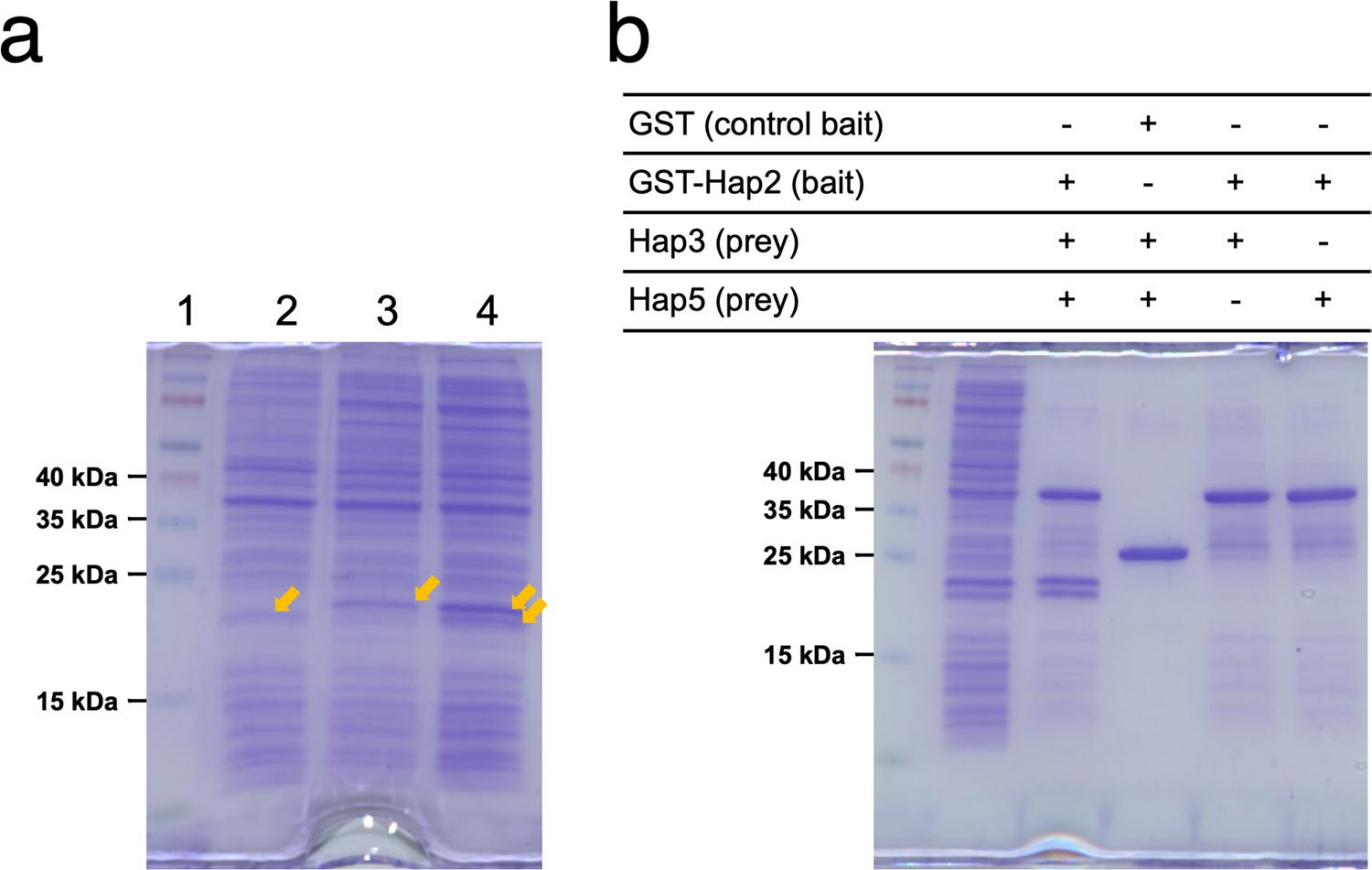
GST-pulldown assay showing physical interaction between *Po*Hap2, *Po*Hap3, and *Po*Hap5. **a** Confirmation of production of prey proteins by SDS-PAGE. Lane 1: molecular weight marker. Lane 2: 10 µl of input sample [lysate of *E*. *coli* BL21 (DE3) cells expressing Hap3 (approximately 17 kDa)] Lane 3: 10 µl of input sample [lysate of *E*. *coli* BL21 (DE3) cells expressing Hap5 (approximately 20 kDa)] Lane 4: 10 µl of input sample [lysate of *E*. *coli* BL21 (DE3) cells expressing both Hap3 and Hap5] **b** SDS-PAGE after GST-pulldown. “ + ” indicates the presence and “ − ” indicates the absence of the respective unpurified recombinant proteins (*E*. *coli* lysate samples). To the right lane of the molecular weight marker, the *E*. *coli* lysate with Hap3 and Hap5, which corresponds to lane 4 of panel **a**, was applied

To determine whether the core region of *Po*Hap2 is also required for the interaction with Hap3 and Hap5, we performed a GST-pulldown assay using recombinant proteins produced in *E*. *coli*: GST-Hap2 (93–173 aa) as bait, and Hap3 (approximately 17 kDa) and Hap5 (approximately 20 kDa) as prey. As shown in Fig. 7, the two prey proteins were pulled down by GST-Hap2, but not by GST. Furthermore, Hap3 or Hap5 were not pulled down when either protein was used as prey. These results show that the core region of Hap2 interacts with Hap3 and Hap5 in vitro*,* but only when both Hap3 and Hap5 are present. Therefore, it is suggested that *Po*Hap2/3/5 form a heterotrimer, as has been reported in other fungi, including Aspergilli (Kato 2005).

**Fig. 8:**
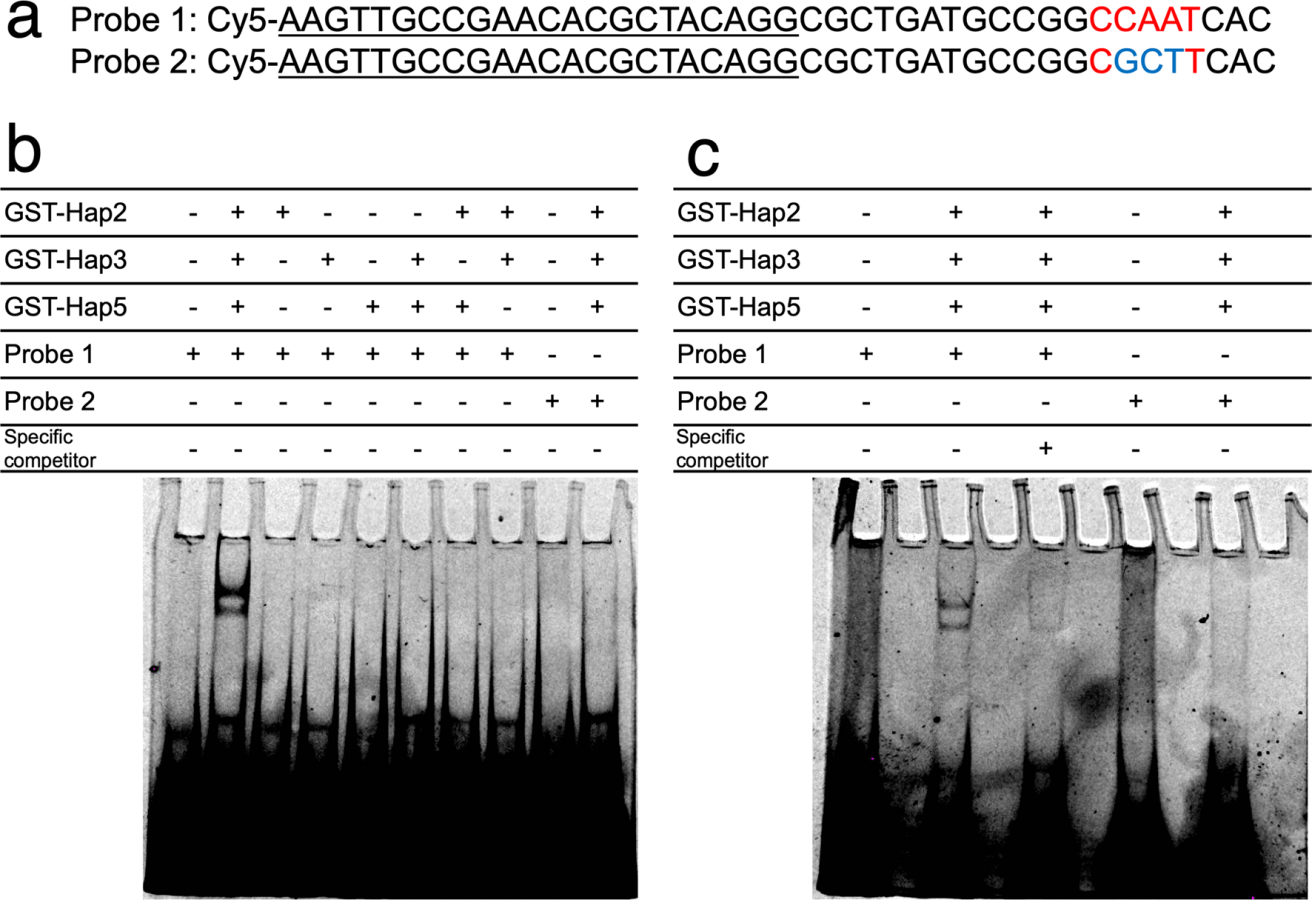
Electrophoretic mobility shift assay (EMSA) showing Hap2/Hap3/Hap5 complex binds to CCAAT sequence in vitro. **a** Nucleotide sequence (5′-3′) of probes used for EMSA. The underlined sequence was used to anneal with the Cy5-labeled oligonucleotide selex_fm2 (Supplemental Table S2). **b** EMSA using 13 pmol each of GST-Hap2 (93–173 aa), GST-Hap3, and/or GST-Hap5. “ + ” indicates the presence and “ − ” indicates the absence of the respective component. **c** EMSA using reduced protein concentrations (2 pmol each). “ + ” indicates the presence and “ − ” indicates the absence of the respective component. Where indicated, the specific competitor (non-labeled) was added at 1600-fold molar excess relative to the labeled probe

The qRT-PCR results suggested that Hap2, potentially Hap complex, plays a crucial role in transcriptional upregulation of *vp2* on lignocellulosic-substrates. However, it was not clarified whether the Hap complex binds directly 5′-upstream of *vp2*. To investigate this, EMSA was performed using recombinant protein samples. Purified GST-Hap2 (aa 93–173), GST-Hap3, and GST-Hap5 were subjected to EMSA with a Cy5-labeled probe containing the sequence 5′-upstream of the *vp2* gene. Probe 1 contained the CCAAT sequence, whereas probe 2 contained a mutation in this sequence (Fig. 8a). As shown in Fig. 8b, two shifted bands were observed for probe 1 only when all the three proteins were present at 13 pmol. In contrast, no shift was observed in probe 2, which contained base substitutions in the CCAAT sequence. This indicates that a multimer composed of GST-Hap2, GST-Hap3, and GST-Hap5 bound to DNA in a CCAAT sequence-specific manner in vitro.

The presence of these two shifted bands can be attributed to the formation of multiple multimeric forms (e.g., tetramers, pentamers, and trimers). To confirm this possibility, EMSA was repeated with a reduced amount of protein (2 pmol each). As shown in Fig. 8c, two shifted bands were still observed for probe 1. In addition, both shifted bands almost disappeared when a specific competitor was added at a 1600–fold molar excess relative to that of the probe. These results suggested that the *P*. *ostreatus* Hap2/Hap3/Hap5 complex specifically binds to the CCAAT sequence.

## Discussion

In this study, we investigated the function of *P*. *ostreatus* Hap, based on the presence of CCAAT sequences in the 5′-upstream regions of *P*. *ostreatus mnp*/*vp* genes. This study was conducted in four main parts: analysis of the expression patterns of *mnp*/*vp* genes in *P*. *ostreatus* strain 20b cultured on four different media, characterization of the effects of *P*. *ostreatus hap2* deletion, examination of in vitro interactions between recombinant *P*. *ostreatus* Hap2, Hap3, and Hap5 to determine their potential for multimer formation, and demonstration of the binding capacity of multimer(s) composed of recombinant GST-Hap2, GST-Hap3, and GST-Hap5 to the CCAAT sequence located 5′-upstream of the *vp2* gene in vitro.

The total transcript copies of *mnp*/*vp* were significantly higher in strain 20b grown on BWS-I, Holocellulose-I, and Avicel-I than that on YMG (Fig. 4a). This result collectively suggests that the presence of lignocellulosic components upregulates expression of *P*. *ostreatus mnp*/*vp* genes. Recent studies on the effects of carbon sources on lignocellulose-degrading enzyme production in *P*. *ostreatus* (Alfaro et al. 2016; Yoav et al. 2018) have shown that carbon catabolite repression likely contributes to lower transcriptional expression or downregulation of *mnp*/*vp* in glucose-containing YMG medium. However, while carbon catabolite repression may contribute to the transcriptional changes observed in this study, additional regulatory mechanisms likely exist, as indicated by the distinct expression pattern observed on Avicel-I compared to BWS-I and Holocellulose-I (Fig. 4b). *mnp*/*vp* transcription may be influenced not only by specific lignocellulosic components and/or nutrients, but also by physical stimulation from the surface of the lignocellulose-based media used in this study.

The results shown in Figs. 6 and 7 suggest that the loss of Hap2 inhibits the mechanisms crucial for the upregulation of *vp2* in lignocellulose-based media. Given that *vp2* transcription is greatly induced by lignocellulosic substrates compared to Avicel (Fig. 4), *vp2* may be specifically upregulated by hemicellulose(s) or an ordered complex of cellulose and hemicellulose.

However, while VP2 (*vp2*) was also predominant when *P*. *ostreatus* was grown on cotton stalks (Salame et al. 2014), and on three different media composed mainly of milled beech wood, rice straw, or Japanese cedar (Nakazawa et al. 2023b), VP2 (*vp2*) was not highly expressed when cultured with other lignocellulosic substrates (Fernández-Fueyo et al. 2014, 2016; Yoav et al. 2018). Therefore, *vp2* is not always predominant when *P*. *ostreatus* is grown on lignocellulosic substrates. This study showed a significant downregulation of not only *vp2* but also other *mnp*/*vp* genes in *hap2* deletants (Figs. 6 and 7, and Supplemental Figs. S2, S3, and S4). The pattern of *mnp*/*vp* genes exhibiting decreased transcript abundance upon *hap2* deletion varied depending on culture conditions. For instance, the significantly reduced transcription of *mnp1* and *mnp6* in BWS-I, but not in Holocellulose-I, was observed in *hap2* deletants. These variabilities suggest that Hap2 (or the HAP complex and CCAAT boxes) participates in the transcriptional regulation of *mnp*/*vp* genes in a context-dependent manner, potentially depending on environmental conditions and other regulatory elements. To gain further insights into the transcriptional regulation mechanisms involving Hap2, future studies are needed to analyze the transcriptional expression patterns of *mnp*/*vp* in *hap2* deletants grown on a broader range of lignocellulose-based substrates, including those on which VP2 (*vp2*) is not predominant. In addition, it is essential to identify and investigate other regulatory elements (transcription factors and chromatin remodelers) that may cooperate with the HAP complex to regulate *mnp*/*vp* transcription.

Previous studies have demonstrated the importance of Hap2 and CCAAT-box elements in the transcriptional regulation of various polysaccharide-degrading enzyme-encoding genes in Aspergilli and *Trichoderma reesei*. These elements play crucial roles in the regulation of amylase-encoding genes in *Aspergillus oryzae* (Brakhage et al. 1999; Tsukagoshi et al. 2001) and cellulose- and xylan-degrading enzyme-encoding genes in *T*. *reesei* (Zeilinger et al. 1998, 2001; Brakhage et al. 1999). In contrast, the result shown in Fig. 3 suggests that unlike *T*. *reesei*, *P*. *ostreatus* Hap2 may not be involved in the transcriptional regulation of cellulolytic and xylanolytic enzyme-encoding genes. Nonetheless, the importance of Hap2 in the positive transcriptional regulation of *mnp*/*vp* in *P*. *ostreatus* indicates that its role is broadly consistent with that in ascomycetous filamentous fungi. In both cases, Hap2 is likely involved in the transcriptional upregulation of genes encoding enzymes crucial for the specific nutrient assimilation strategies and lifestyles of each fungus. This divergence in the regulatory targets can be understood in the context of fungal evolution. White-rot fungi, including *P*. *ostreatus*, have acquired lignin (or lignocellulose)-degrading abilities over their evolutionary history. This acquisition allows them to adopt a lifestyle distinct from that of other fungi, including ascomycetes, which primarily utilize polysaccharides found in simpler substrates. Consequently, it is plausible that the target enzyme-encoding genes regulated by the HAP complex were altered during this evolutionary transition, reflecting the specialized degradative capabilities of white-rot fungi.

In conclusion, the results of this study suggest that *P*. *ostreatus* Hap2 plays a significant role in *vp2* transcription in lignocellulose-based media. In vitro experiments have also suggested that *P*. *ostreatus* Hap2, Hap3, and Hap5 form a trimeric complex that binds to the DNA fragment containing the CCAAT sequence, similar to that in humans, *A*. *thaliana*, and *A*. *nidulans* (Nardone et al. 2017). However, this DNA–protein interaction has not been confirmed in vivo*.* Consequently, it remains unclear whether the CCAAT sequence present in the 5′-upstream sequence of *vp2* is crucial for its high expression in lignocellulose-based media. To address this question, we are currently investigating the effect of mutations in the CCAAT sequence of 5′-upstream of *vp2* on its transcript level. Our initial approach involved the introduction of mutations in the CCAAT sequence by homologous recombination with the *hph* gene. However, no clear or stable difference in *vp2* transcription levels was observed between the strains with mutated and unmutated CCAAT sequences 5′-upstream of *vp2*. In addition, the insertion of *hph* into the 5′-upstream region itself negatively affected *vp2* transcript levels (data not shown). These results suggest two possibilities: either the CCAAT sequence 5′-upstream of *vp2* may not be crucial for *vp2* transcription, or the function of the CCAAT sequence and Hap complex is impaired by mechanisms such as transcriptional interference caused by the *hph* insertion. To properly investigate the role of the CCAAT sequence upstream of *vp2*, it is necessary to introduce mutations without inserting *hph*. This may be achieved by genome editing using transient transformation, a technique previously developed in our laboratory (Koshi et al. 2022). Future studies focusing on these aspects will provide more definitive insights into the role of the hypothetical *P. ostreatus* HAP complex in the upregulation of *vp2* transcription.

## Supplementary Information

Below is the link to the electronic supplementary material.

Supplementary file1 (PDF 443 KB)

## Data Availability

All data supporting the claims of this manuscript are presented and made available in this manuscript and Supplemental Information.
