# Supplementary material for: Effects of hap2 deletion on mnp/vp transcription in Pleurotus ostreatus grown on lignocellulosic substrates
Source: Appl Microbiol Biotechnol. 2024 Nov 13;108:513. doi: 10.1007/s00253-024-13352-7 (PMC11561020; doi:10.1007/s00253-024-13352-7)
Supplement: Supplementary file 1 [file 253_2024_13352_MOESM1_ESM.pdf]

Supplementary file

Applied Microbiology and biotechnology

Effects of *hap2* deletion on *mnp*/*vp* transcription in *Pleurotus ostreatus* grown on lignocellulosic substrates

Keita Kayama, Takehito Nakazawa*, Iori Yamaguchi, Moriyuki Kawauchi, Masahiro Sakamoto, Yoichi Honda

Graduate School of Agriculture, Kyoto University, Sakyo-ku, Kyoto 606-8502, Japan

*Corresponding author: Takehito Nakazawa

Graduate School of Agriculture, Kyoto University, Oiwakecho, Kitashirakawa,

Sakyo-ku, Kyoto 606-8502, Japan

Tel.: +81 75 753 6465

Fax: +81 75 753 6471

E-mail: nakazawa.takehito.8u@kyoto-u.ac.jp

Running Title: Functional analysis of *P. ostreatus* hap2

Table S1 The composition of lignocellulose-based media used in this study.

Media	Composition
BWS-I	1.9 g extracted[a] beech wood sawdust, 0.1 g extracted[a] wheat bran, and 6.0 ml water
BWS-II	1.9 g size-fractionated beech wood sawdust (250–500 μm), 0.1 g wheat bran, and 6.0 ml water
Avicel-I	1.9 g Avicel[b], 0.1 g extracted[a] wheat bran, and 6.0 ml water
Holocellulose-I	1.9 g holocellulose[c], 0.1 g extracted[a] wheat bran, and 6.0 ml water

[a]Solvent extraction was performed as described by Nakazawa et al. (2023a).
[b]Avicel is a crystalline cellulose with an average degree of 200.
[c]Holocellulose was prepared by the Jayme-Wise method (Green 1963) as described by Nakazawa et al. (2023a).

Table S2 Primers used for GST-pulldown, *hap2* deletion, and EMSA

Primer	Sequence（5′→3′）
KYK136	GTGATTGGCCGGCATCAGCG
KYK137	CGCTGATGCCGGCCAATCAC
M13R	ACAATTTCACACAGGAAACAGCTATGACC
Selex_R	GCACGATGACAGCATACTCTAAG
Selex_Rdm_comp	NNNNNNNNNNNNNNNNNNNNGCTTAGAGTATGCTGTCATCGTGC
TN48	TGGCTGTTGACTTATCGTGGCG
TN378	TCACAAGTTAACAGCCACGGATTAAAAGC
TN400	TCCCAGTCACGACGTCAGGCAAAACCCAGATACGC
TN991	TCGGGGTCCTGCATGCTAGCG
TN992	ACGTCGTGACTGGGATCTATGGGGGCCTCGTCTGG
TN993	CCTGTGTGAAATTGTATCGACCCTACTGCCCCGCC
TN994	TCCCACCGCCGGACGTTTAC
TN995	ATCGACAGGGGCTGACCTCTTCTG
TN996	AGCGCGACAGCAATGTAGGG
TN997	AGCAGTTCCACCCGCATCAG
TN998	TGGCTGCATGTTCATTGGTGG
TN1097	CGGCCGCATCGTGACCCAGACGAGGCCCCCATAGAC
TN1098	GCAGATCGTCAGTCAAGTAGGGTCGATGCCCGCTG
TN1523	CATATGTATATCTCCTTATTGTTTC
TN1524	CATACCAAGCGGGCCCTGAAAC
TN1526	TAATGACGTACCATGGTAAAGTCTACACC
TN1528	GGAGATATACATATGATGTCCGATAGCCTCCAG
TN1529	CATGGTACGTCATTATTCTTCATCTAACGACTCTAGC
TN1531	GGAGATATACATATGTCGTCCAGCAAGCCTTTCGTC
TN1532	CATGGTACGTCATTAAGAAGGGATGTTATTCCGTTCTG
TNS12	GTGATTGGCCGGCATCAGCGCCTGTAGCGTGTTCGGCAACTT
TNS13	GTGAAGCGCCGGCATCAGCGCCTGTAGCGTGTTCGGCAACTT

Table S3 Primers used for the real-time PCR and digital PCR.

Target gene (Protein ID[a])	Sequence (5′ - 3′)	Amplification efficiency (%)
β-tub[b]	GTGCGTAAGGAAGCTGAGGG<br>TGTGGCATTGTACGGCTCAAC	98
vp1[b]	TTGTTGGCTAGAGACCCCCAGA<br>CAAGTGGGCCGCTCCGAC	83.5
vp2[b]	GCCTTTCGATAGCGTGGATAAG<br>GGTCCCTTGCAACATTGTCTC	95.1
vp3[b]	CAGGCCGTCAATGCCGTA<br>CGTAAGGACGGAACCATCA	104.7
mnp1[b]	GTTCGCTCGAGACGATAGGACAT<br>GGGGAGATGTGGTTTGGTTACA	95.2
mnp2[b]	ATTGCTAATGAAAGGCTCATGGTT<br>GGTGGCAGCACAAGCAG	102.9
mnp3[b]	CCTCCTGACTTTGGCATCTCA<br>TGTCGTCGATTCCACCGTTG	82.5
mnp4[b]	CTTCATCTCCAACCCCAACTC<br>GCTTTGCGGCAGGATG	107.1
mnp5[b]	TTCCCAGGTACTGCCGGA<br>CCAAAGACAGTTTCAACATCGC	117.6
mnp6[b]	CTTCGACTCTACTCCCAACAGC<br>GAGGCGTGGTCGGTGAT	94

[a]A genomic fragment containing the genes that corresponds to each Protein ID were from the genome database of strain PC9 (JGI *Pleurotus ostreatus* PC9 v1.0, https://genome.jgi.doe.gov/PleosPC9_1/PleosPC9_1.home.html).

[b]Primers designed in Salame et al. (2012b)

Table S4 Probes used for digital PCR.

Gene[a]	Sequence (5´- 3´)[b]	PCR condition[c]
β-tubulin	TGCTGGTATGGGTACACTCCTGAT	A
vp1	CAGTCCATGGTTAACAACCAGC	A
vp2	ACCGTTGAAGTCGTCTGGCTGC	A
vp3	AGCGAATAGGCGCAATGCATTTC	A
mnp1	ACCTTCAGCAGAACCTCTTCGACG	A
mnp2	CAGGACGCCATGATCGATTG	A
mnp3	AGCCACCCTTGGACAAGTCC	B
mnp4	CTCTTTGATGGTGCCGAGTGTGG	A
mnp5	AACCCAAAGAAGCTGATTGACTGC	B
mnp6	CGAGGAGAGATGCGGCTTCAGTC	A

[a] Probes for the indicated genes were originally designated by Nakazawa et al. (2023b).

[b] The synthesized oligonucleotides are modified with 6-FAM (5´), ZEM quencher (internal) and lowa Black Dark quencher (3´).

[c] The PCR condition was as follows: one cycle at 95°C (10 min) for initial denaturation, 40 cycles at 95°C (30 sec) and 58°C (A) or 61°C (B) (60 sec), followed by denature step at 95°C (10 min). Ramp Rate was 2°C/sec.

Table S5 Promoter analysis of *G. subvermispora*

Gene[a]	protein ID[b]	TATAA-box[c, d]	CCAAT-box[c, e, f]
gp	112162	-	-
lip1	99382	-	160(-)
lip2	118677	67	167(-)
mnp1	116608	90	149(-)
mnp2	50297	95	-
mnp3	139965	98, 89	148(-)
mnp4	94398	83	163(-)
mnp5	49863	93	175(-)
mnp6	50686	95	297(+)
mnp7	105539	75	230(-), 146(-), 106(-)
mnp8	114036	91	161(-)
mnp9	114076	-	-
mnp10	117436	94	238(+), 172(-), 46(+)
mnp11	143390	78	149(-)
mnp12	157986	-	-
mnp13	124076	76	186(-)
lcs1	118801	-	-
lcs2	88089	-	-
lcs3	108852	-	75(-)
lcs4	137686	76	-
lcs5	115068	83	201(+)
lcs6	115063	74	243(+)
lcs7	84170	82	240(+)

[a, b] Gene names and protein ID are based on the genome database of *Gelatoporia subvermispora* B (https://mycocosm.jgi.doe.gov/Cersu1/Cersu1.home.html)

[c] The distances of the TATAA-box and CCAAT-box from the start codon are shown.

[d] Only TATAA-boxes located within 100 bp from the start codon are shown.

[e] CCAAT-boxes located within 300 bp from the start codon are shown.

[f] (+) indicates the presence of CCAAT on the coding strand, while (-) on the template strand.

Table S6 Promoter analysis of *P. chrysosporium*

Gene[a]	protein ID[b]	TATAA-box[c, d]	CCAAT-box[c, e, f]
lipA	2989894	81	180(-)
lipB	1716776	77	258(+)
lipC	3032409	81	-
lipD	1386770	88	192(-)
lipE	1716042	77	-
lipF	2910310	74	166(-)
lipG	2918435	79	-
lipH	2918661	71	-
lipI	3032425	84	188(-)
lipJ	3043032	71	193(-)
mnp1	2971944	90	191(-), 205(-)
mnp2	3589	87	236(-)
mnp3	2896529	-	-
mnp4	8191	93	194(-), 208(-)
mnp5	2907883	-	19(+), 153(-)

[a, b] Gene names and protein ID are based on the genome database of *Phanerochaete chrysosporium* RP-78 (https://mycocosm.jgi.doe.gov/Phchr2/Phchr2.home.html)

[c] The distances of the TATAA-box and CCAAT-box from the start codon are shown.

[d] Only TATAA-boxes located within 100 bp from the start codon are shown.

[e] CCAAT-boxes located within 300 bp from the start codon are shown.

[f] (+) indicates the presence of CCAAT on the coding strand, while (-) on the template strand.

Table S7 Homology search against CBC (CCAAT-binding complex) components of *Aspergillus nidulans*.

Query (*Aspergillus nidulans*)		1st Hit (*Pleurotus ostreatus*)				2nd Hit (*Pleurotus ostreatus*)			
Protein name	NCBI Accession no.	NCBI Accession no.	Query cover	Percent Identification	E-value	NCBI Accession no.	Query cover	Percent Identification	E-value
HapB	XP_050467982.1	XP_036630745.1	26%	62.63%	3.00E-28	-	-	-	-
HapC	XP_661638.1	XP_036626823.1	42%	73.12%	2.00E-47	XP_036633301.1	48%	33.96%	6.00E-17
HapE	XP_664096.2	XP_036633731.1	39%	65.71%	7.00E-47	-	-	-	-

Table S8 Reciprocal Blastp for identification of CBC (CCAAT-binding complex) components.

Query (*Pleurotus ostreatus*)		1st Hit (*Aspergillus nidulans*)				2nd Hit (*Aspergillus nidulans*)			
Protein name	NCBI Accession no.	NCBI Accession no.	Query cover	Percent Identification	E-value	NCBI Accession no.	Query cover	Percent Identification	E-value
Putative Hap2	XP_036630745.1	XP_050467982.1	22%	64.21%	3.00E-28	-	-	-	-
Putative Hap3	XP_036626823.1	XP_661638.1	61%	71.13%	2.00E-47	XP_050468969.1	47%	38.46%	9.00E-12
Putative Hap5	XP_036633731.1	XP_664096.2	56%	65.71%	6.00E-47	-	-	-	-

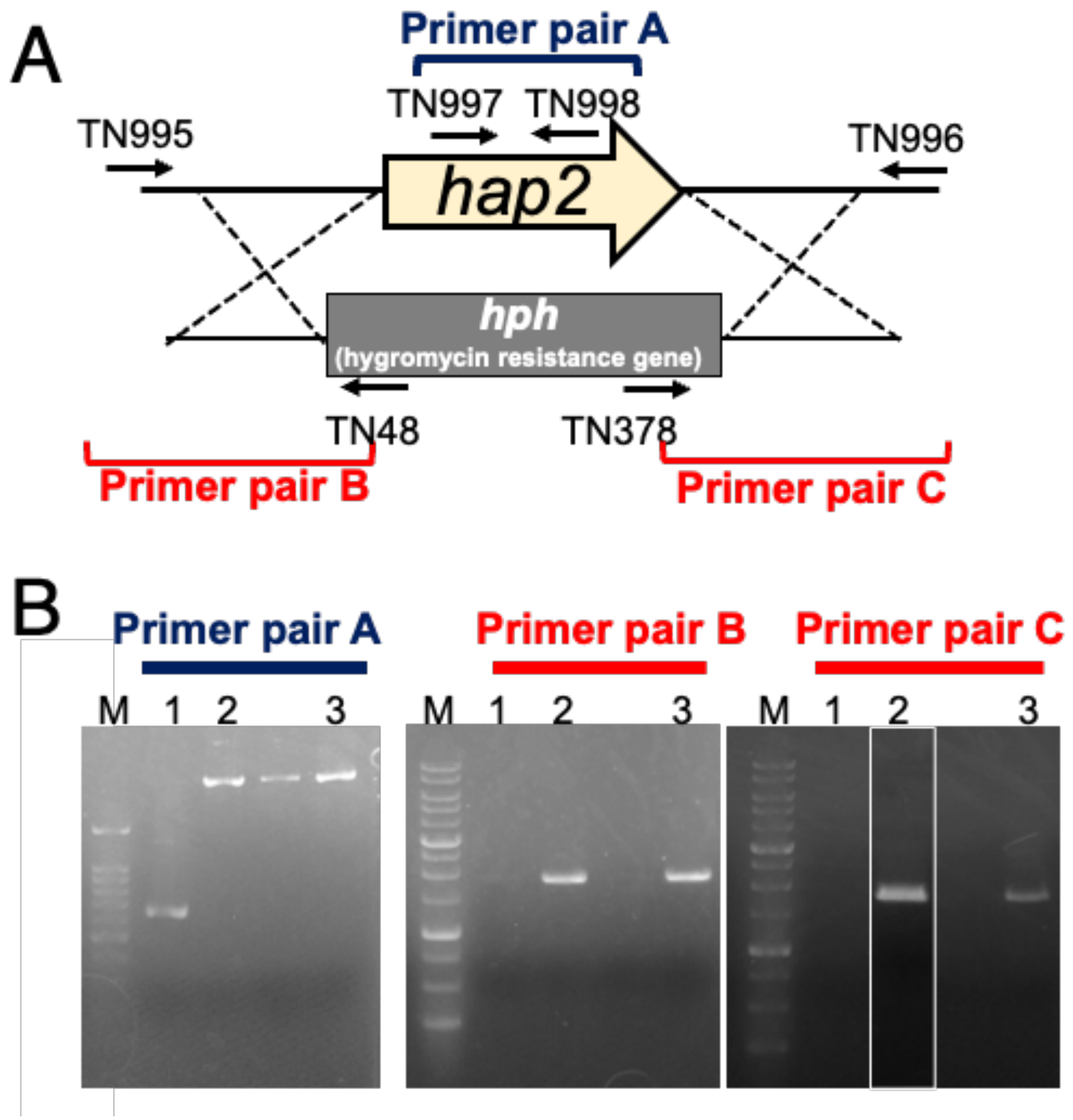

Fig. S1 Generation of the *hap2* deletants (Δ*hap2*#1 and Δ*hap2*#2) derived from 20b using homologous recombination. (A) A diagram of the genomic locus of *Pleurotus ostreatus hap2*. Black arrows indicate the primers used for the polymerase chain reaction (PCR) amplification. (B) Genomic PCR experiments confirming the *hap2* deletion in the deletants. Primers used in each PCR experiment are shown in (A).

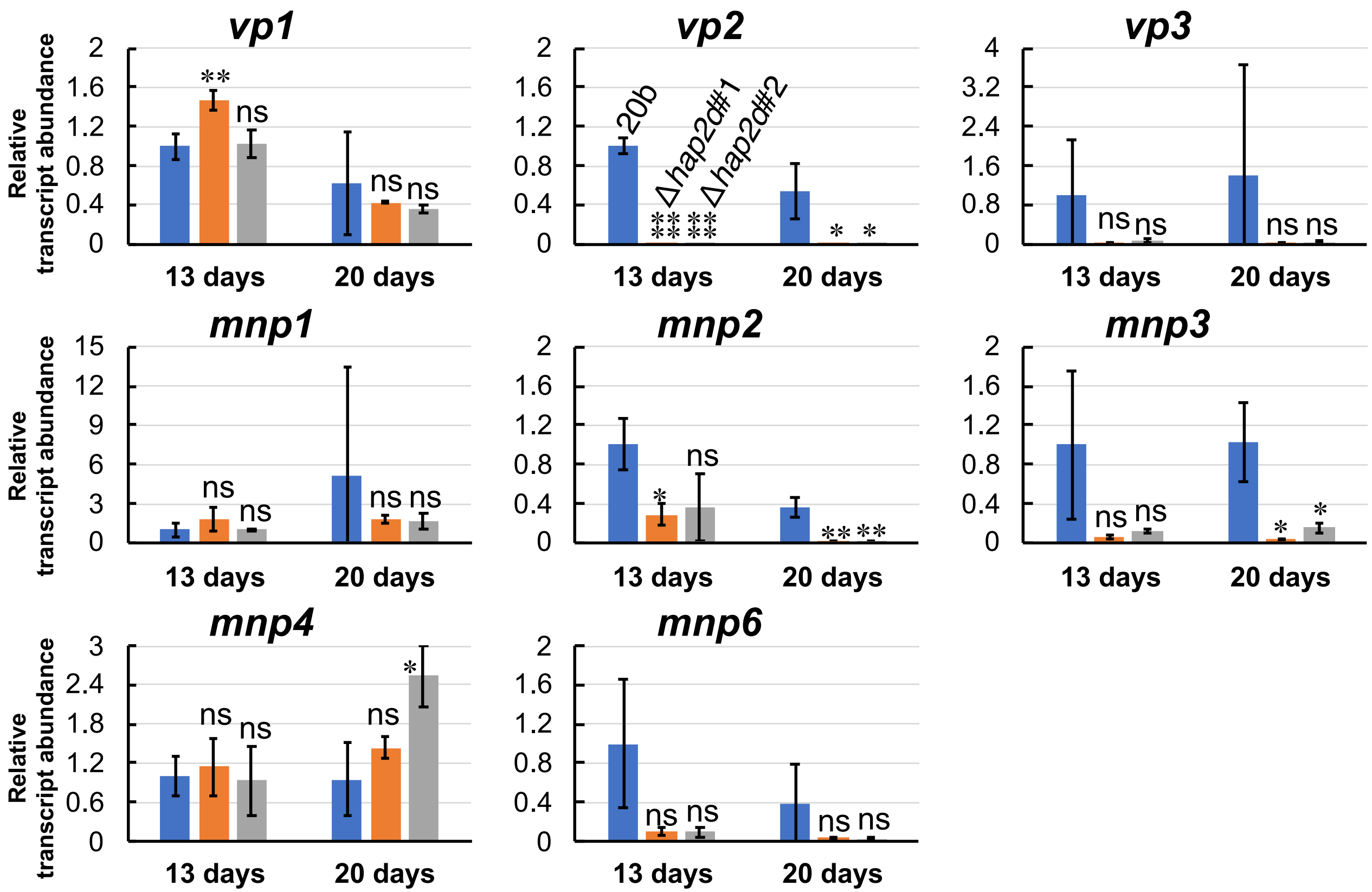

Fig. S2 Relative transcriptional expression levels of the eight *mnp*/*vp* genes in the *hap2* deletants grown on Holocellulosel-I at 13- and 20-day culture periods (n = 3). Expression levels were standardized by *β-tubulin*, and the expression level of 20b at 13 days is shown as 1. Graphs indicate mean values and bars indicate standard deviations. Statistical significance tests between the indicated two strains at the respective culture periods were performed using a two-tailed equal variance *t*-test. *$p < 0.05$, **$p < 0.01$, ***$p < 0.001$. "ns" indicates statistical non-significance ($p > 0.05$).

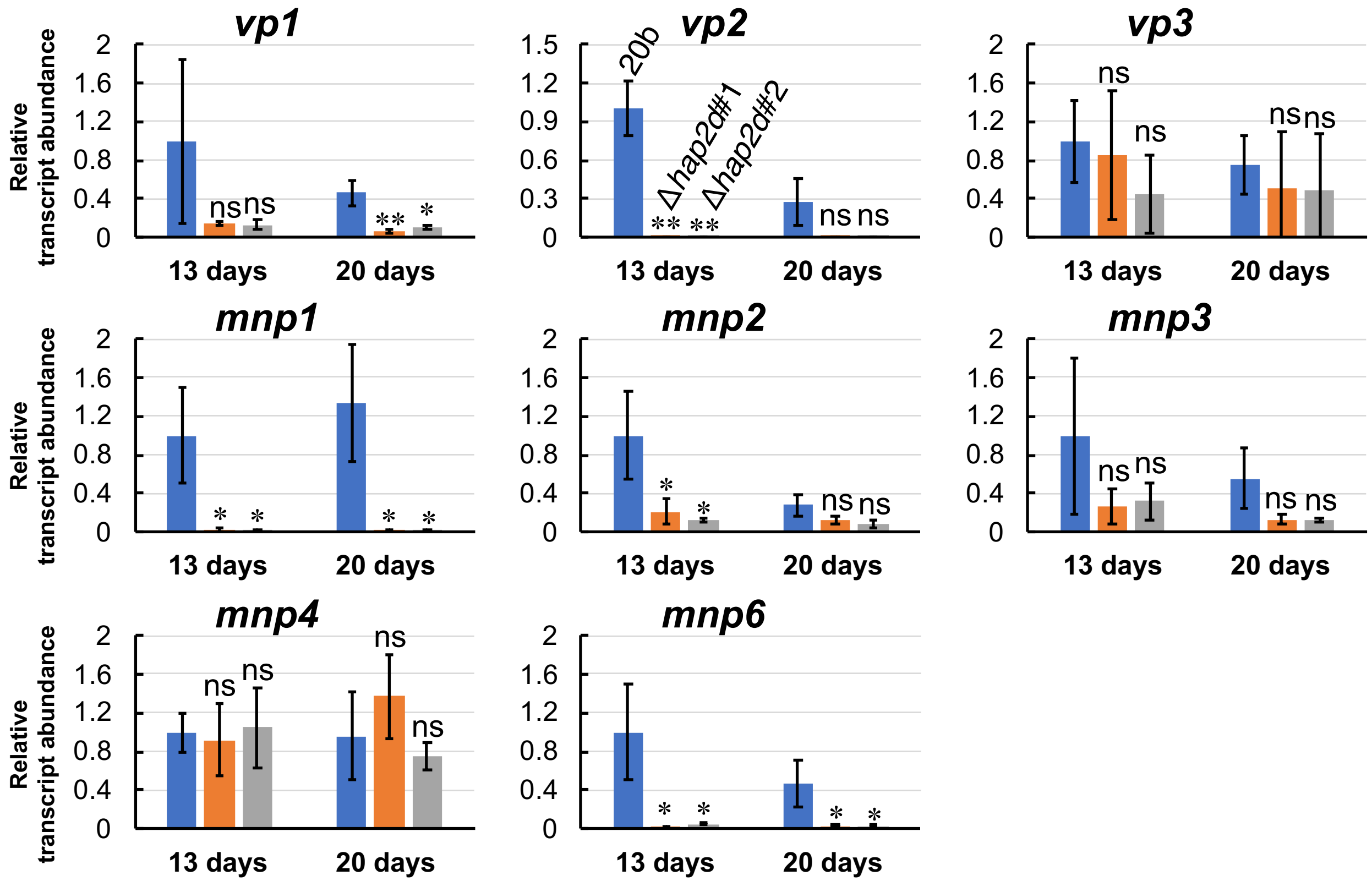

Fig. S3 Relative transcriptional expression levels of the eight *mnp*/*vp* genes in the *hap2* deletants grown on Avicel-I at 13- and 20-day culture periods (n = 3). Expression levels were standardized by *β-tubulin*, and the expression level of 20b at 13 days is shown as 1. Graphs indicate mean values and bars indicate standard deviations. Statistical significance tests between the indicated two strains at the respective culture periods were performed using a two-tailed equal variance *t*-test. $*p < 0.05$, $**p < 0.01$, $***p < 0.001$. "ns" indicates statistical non-significance ($p > 0.05$).

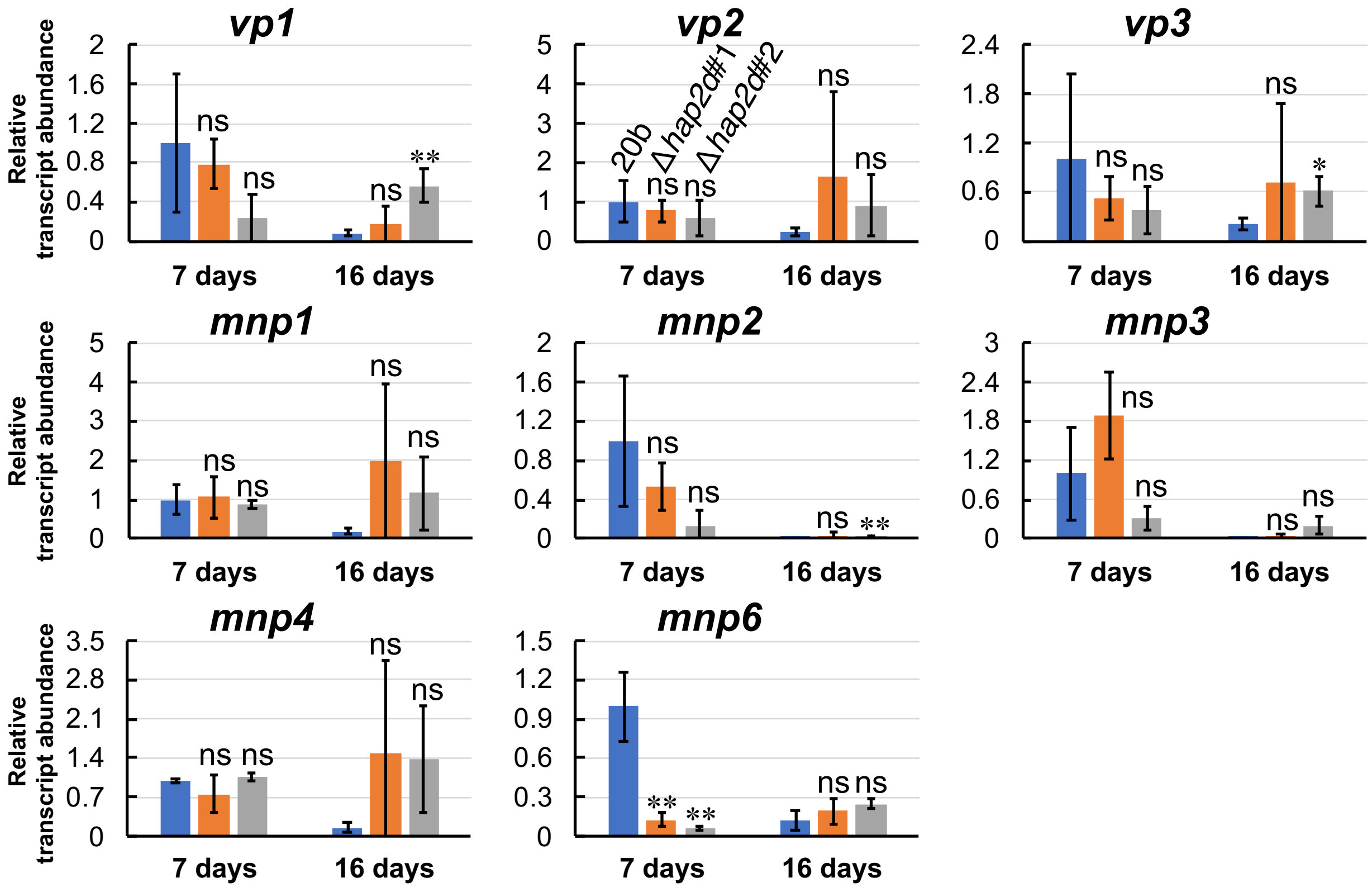

Fig. S4 Relative transcriptional expression levels of the eight *mnp*/*vp* genes in the *hap2* deletants grown on YMG agar plates at 7- and 16-day culture periods (n = 3). Expression levels were standardized by *β-tubulin*, and the expression level of 20b at 7 days is shown as 1. Graphs indicate mean values and bars indicate standard deviations. Statistical significance tests between the indicated two strains at the respective culture periods were performed using a two-tailed equal variance *t*-test. *$p < 0.05$, **$p < 0.01$, ***$p < 0.001$. “ns” indicates statistical non-significance ($p > 0.05$).
