# Supplementary material for: Effects of hap2 deletion on mnp/vp transcription in Pleurotus ostreatus grown on lignocellulosic substrates
Source: Appl Microbiol Biotechnol. 2024 Nov 13;108(1):513. doi: 10.1007/s00253-024-13352-7 (PMC11561020; doi:10.1007/s00253-024-13352-7)
Supplement: Supplementary file 1 — Supplementary file1 (PDF 443 KB) [file 253_2024_13352_MOESM1_ESM.pdf]

Supplementary file

Applied Microbiology and biotechnology

**Effects of *hap2* deletion on *mnp*/*vp* transcription in *Pleurotus ostreatus* grown on lignocellulosic substrates**

**Keita Kayama, Takehito Nakazawa*, Iori Yamaguchi, Moriyuki Kawauchi, Masahiro Sakamoto, Yoichi Honda**

Graduate School of Agriculture, Kyoto University, Sakyo-ku, Kyoto 606-8502, Japan

*Corresponding author: Takehito Nakazawa

Graduate School of Agriculture, Kyoto University, Oiwakecho, Kitashirakawa, Sakyo-ku, Kyoto 606-8502, Japan

Tel.: +81 75 753 6465

Fax: +81 75 753 6471

E-mail: nakazawa.takehito.8u@kyoto-u.ac.jp

Running Title: Functional analysis of *P. ostreatus* hap2

**Table S1** The composition of lignocellulose-based media used in this study.

| Media | Composition |
|---|---|
| BWS-I | 1.9 g extracted[a] beech wood sawdust, 0.1 g extracted[a] wheat bran, and 6.0 ml water |
| BWS-II | 1.9 g size-fractionated beech wood sawdust (250–500 μm), 0.1 g wheat bran, and 6.0 ml water |
| Avicel-I | 1.9 g Avicel[b], 0.1 g extracted[a] wheat bran, and 6.0 ml water |
| Holocellulose-I | 1.9 g holocellulose[c], 0.1 g extracted[a] wheat bran, and 6.0 ml water |

[a]Solvent extraction was performed as described by Nakazawa et al. (2023a).

[b]Avicel is a crystalline cellulose with an average degree of 200.

[c]Holocellulose was prepared by the Jayme-Wise method (Green 1963) as described by Nakazawa et al. (2023a).

**Table S2** Primers used for GST-pulldown, *hap2* deletion, and EMSA

| Primer | Sequence（5′→3′） |
|---|---|
| KYK136 | GTGATTGGCCGGCATCAGCG |
| KYK137 | CGCTGATGCCGGCCAATCAC |
| M13R | ACAATTTCACACAGGAAACAGCTATGACC |
| Selex_R | GCACGATGACAGCATACTCTAAG |
| Selex_Rdm_comp | NNNNNNNNNNNNNNNNNNNNGCTTAGAGTATGCTGTCATCGTGC |
| TN48 | TGGCTGTTGACTTATCGTGGCG |
| TN378 | TCACAAGTTAACAGCCACGGATTAAAAGC |
| TN400 | TCCCAGTCACGACGTCAGGCAAAACCCAGATACGC |
| TN991 | TCGGGGTCCTGCATGCTAGCG |
| TN992 | ACGTCGTGACTGGGATCTATGGGGGCCTCGTCTGG |
| TN993 | CCTGTGTGAAATTGTATCGACCCTACTGCCCCGCC |
| TN994 | TCCCACCGCCGGACGTTTAC |
| TN995 | ATCGACAGGGGCTGACCTCTTCTG |
| TN996 | AGCGCGACAGCAATGTAGGG |
| TN997 | AGCAGTTCCACCCGCATCAG |
| TN998 | TGGCTGCATGTTCATTGGTGG |
| TN1097 | CGGCCGCATCGTGACCCAGACGAGGCCCCCATAGAC |
| TN1098 | GCAGATCGTCAGTCAAGTAGGGTCGATGCCCGCTG |
| TN1523 | CATATGTATATCTCCTTATTGTTTC |
| TN1524 | CATACCAAGCGGGCCCTGAAAC |
| TN1526 | TAATGACGTACCATGGTAAAGTCTACACC |
| TN1528 | GGAGATATACATATGATGTCCGATAGCCTCCAG |
| TN1529 | CATGGTACGTCATTATTCTTCATCTAACGACTCTAGC |
| TN1531 | GGAGATATACATATGTCGTCCAGCAAGCCTTTCGTC |
| TN1532 | CATGGTACGTCATTAAGAAGGGATGTTATTCCGTTCTG |
| TNS12 | GTGATTGGCCGGCATCAGCGCCTGTAGCGTGTTCGGCAACTT |
| TNS13 | GTGAAGCGCCGGCATCAGCGCCTGTAGCGTGTTCGGCAACTT |

**Table S3** Primers used for the real-time PCR and digital PCR.

| Target gene (Protein ID[a]) | Sequence (5′ - 3′) | Amplification efficiency (%) |
|---|---|---|
| *β-tub*[b] | GTGCGTAAGGAAGCTGAGGG<br>TGTGGCATTGTACGGCTCAAC | 98 |
| *vp1*[b] | TTGTTGGCTAGAGACCCCCAGA<br>CAAGTGGGCCGCTCCGAC | 83.5 |
| *vp2*[b] | GCCTTTCGATAGCGTGGATAAG<br>GGTCCCTTGCAACATTGTCTC | 95.1 |
| *vp3*[b] | CAGGCCGTCAATGCCGTA<br>CGTAAGGACGGAACCATCA | 104.7 |
| *mnp1*[b] | GTTCGCTCGAGACGATAGGACAT<br>GGGGAGATGTGGTTTGGTTACA | 95.2 |
| *mnp2*[b] | ATTGCTAATGAAAGGCTCATGGTT<br>GGTGGCAGCACAAGCAG | 102.9 |
| *mnp3*[b] | CCTCCTGACTTTGGCATCTCA<br>TGTCGTCGATTCCACCGTTG | 82.5 |
| *mnp4*[b] | CTTCATCTCCAACCCCAACTC<br>GCTTTGCGGCAGGATG | 107.1 |
| *mnp5*[b] | TTCCCAGGTACTGCCGGA<br>CCAAAGACAGTTTCAACATCGC | 117.6 |
| *mnp6*[b] | CTTCGACTCTACTCCCAACAGC<br>GAGGCGTGGTCGGTGAT | 94 |

[a]A genomic fragment containing the genes that corresponds to each Protein ID were from the genome database of strain PC9 (JGI *Pleurotus ostreatus* PC9 v1.0, https://genome.jgi.doe.gov/PleosPC9_1/PleosPC9_1.home.html).

[b]Primers designed in Salame et al. (2012b)

**Table S4** Probes used for digital PCR.

| Gene[a] | Sequence (5′- 3′)[b] | PCR condition[c] |
|---|---|---|
| *β-tubulin* | TGCTGGTATGGGTACACTCCTGAT | A |
| *vp1* | CAGTCCATGGTTAACAACCAGC | A |
| *vp2* | ACCGTTGAAGTCGTCTGGCTGC | A |
| *vp3* | AGCGAATAGGCGCAATGCATTTC | A |
| *mnp1* | ACCTTCAGCAGAACCTCTTCGACG | A |
| *mnp2* | CAGGACGCCATGATCGATTG | A |
| *mnp3* | AGCCACCCTTGGACAAGTCC | B |
| *mnp4* | CTCTTTGATGGTGCCGAGTGTGG | A |
| *mnp5* | AACCCAAAGAAGCTGATTGACTGC | B |
| *mnp6* | CGAGGAGAGATGCGGCTTCAGTC | A |

[a] Probes for the indicated genes were originally designated by Nakazawa et al. (2023b).

[b] The synthesized oligonucleotides are modified with 6-FAM (5′), ZEM quencher (internal) and lowa Black Dark quencher (3′).

[c] The PCR condition was as follows: one cycle at 95°C (10 min) for initial denaturation, 40 cycles at 95°C (30 sec) and 58°C (A) or 61°C (B) (60 sec), followed by denature step at 95°C (10 min). Ramp Rate was 2°C/sec.

**Table S5** Promoter analysis of *G. subvermispora*

| Gene[a] | protein ID[b] | TATAA-box[c, d] | CCAAT-box[c, e, f] |
|---|---|---|---|
| *gp* | 112162 | - | - |
| *lip1* | 99382 | - | 160(-) |
| *lip2* | 118677 | 67 | 167(-) |
| *mnp1* | 116608 | 90 | 149(-) |
| *mnp2* | 50297 | 95 | - |
| *mnp3* | 139965 | 98, 89 | 148(-) |
| *mnp4* | 94398 | 83 | 163(-) |
| *mnp5* | 49863 | 93 | 175(-) |
| *mnp6* | 50686 | 95 | 297(+) |
| *mnp7* | 105539 | 75 | 230(-), 146(-), 106(-) |
| *mnp8* | 114036 | 91 | 161(-) |
| *mnp9* | 114076 | - | - |
| *mnp10* | 117436 | 94 | 238(+), 172(-), 46(+) |
| *mnp11* | 143390 | 78 | 149(-) |
| *mnp12* | 157986 | - | - |
| *mnp13* | 124076 | 76 | 186(-) |
| *lcs1* | 118801 | - | - |
| *lcs2* | 88089 | - | - |
| *lcs3* | 108852 | - | 75(-) |
| *lcs4* | 137686 | 76 | - |
| *lcs5* | 115068 | 83 | 201(+) |
| *lcs6* | 115063 | 74 | 243(+) |
| *lcs7* | 84170 | 82 | 240(+) |

[a, b] Gene names and protein ID are based on the genome database of *Gelatoporia subvermispora* B (https://mycocosm.jgi.doe.gov/Cersu1/Cersu1.home.html)

[c] The distances of the TATAA-box and CCAAT-box from the start codon are shown.

[d] Only TATAA-boxes located within 100 bp from the start codon are shown.

[e] CCAAT-boxes located within 300 bp from the start codon are shown.

[f] (+) indicates the presence of CCAAT on the coding strand, while (-) on the template strand.

**Table S6** Promoter analysis of *P. chrysosporium*

| Gene[a] | protein ID[b] | TATAA-box[c, d] | CCAAT-box[c, e, f] |
|---|---|---|---|
| *lipA* | 2989894 | 81 | 180(-) |
| *lipB* | 1716776 | 77 | 258(+) |
| *lipC* | 3032409 | 81 | - |
| *lipD* | 1386770 | 88 | 192(-) |
| *lipE* | 1716042 | 77 | - |
| *lipF* | 2910310 | 74 | 166(-) |
| *lipG* | 2918435 | 79 | - |
| *lipH* | 2918661 | 71 | - |
| *lipI* | 3032425 | 84 | 188(-) |
| *lipJ* | 3043032 | 71 | 193(-) |
| *mnp1* | 2971944 | 90 | 191(-), 205(-) |
| *mnp2* | 3589 | 87 | 236(-) |
| *mnp3* | 2896529 | - | - |
| *mnp4* | 8191 | 93 | 194(-), 208(-) |
| *mnp5* | 2907883 | - | 19(+), 153(-) |

[a, b] Gene names and protein ID are based on the genome database of *Phanerochaete chrysosporium* RP-78 (https://mycocosm.jgi.doe.gov/Phchr2/Phchr2.home.html)

[c] The distances of the TATAA-box and CCAAT-box from the start codon are shown.

[d] Only TATAA-boxes located within 100 bp from the start codon are shown.

[e] CCAAT-boxes located within 300 bp from the start codon are shown.

[f] (+) indicates the presence of CCAAT on the coding strand, while (-) on the template strand.

**Table S7** Homology search against CBC (CCAAT-binding complex) components of *Aspergillus nidulans*.

| Query (*Aspergillus nidulans*) | | 1st Hit (*Pleurotus ostreatus*) | | | | 2nd Hit (*Pleurotus ostreatus*) | | | |
|---|---|---|---|---|---|---|---|---|---|
| Protein name | NCBI Accession no. | NCBI Accession no. | Query cover | Percent Identification | E-value | NCBI Accession no. | Query cover | Percent Identification | E-value |
| HapB | XP_050467982.1 | XP_036630745.1 | 26% | 62.63% | 3.00E-28 | - | - | - | - |
| HapC | XP_661638.1 | XP_036626823.1 | 42% | 73.12% | 2.00E-47 | XP_036633301.1 | 48% | 33.96% | 6.00E-17 |
| HapE | XP_664096.2 | XP_036633731.1 | 39% | 65.71% | 7.00E-47 | - | - | - | - |

**Table S8** Reciprocal Blastp for identification of CBC (CCAAT-binding complex) components.

| Query (*Pleurotus ostreatus*) | | 1st Hit (*Aspergillus nidulans*) | | | | 2nd Hit (*Aspergillus nidulans*) | | | |
|---|---|---|---|---|---|---|---|---|---|
| Protein name | NCBI Accession no. | NCBI Accession no. | Query cover | Percent Identification | E-value | NCBI Accession no. | Query cover | Percent Identification | E-value |
| Putative Hap2 | XP_036630745.1 | XP_050467982.1 | 22% | 64.21% | 3.00E-28 | - | - | - | - |
| Putative Hap3 | XP_036626823.1 | XP_661638.1 | 61% | 71.13% | 2.00E-47 | XP_050468969.1 | 47% | 38.46% | 9.00E-12 |
| Putative Hap5 | XP_036633731.1 | XP_664096.2 | 56% | 65.71% | 6.00E-47 | - | - | - | - |

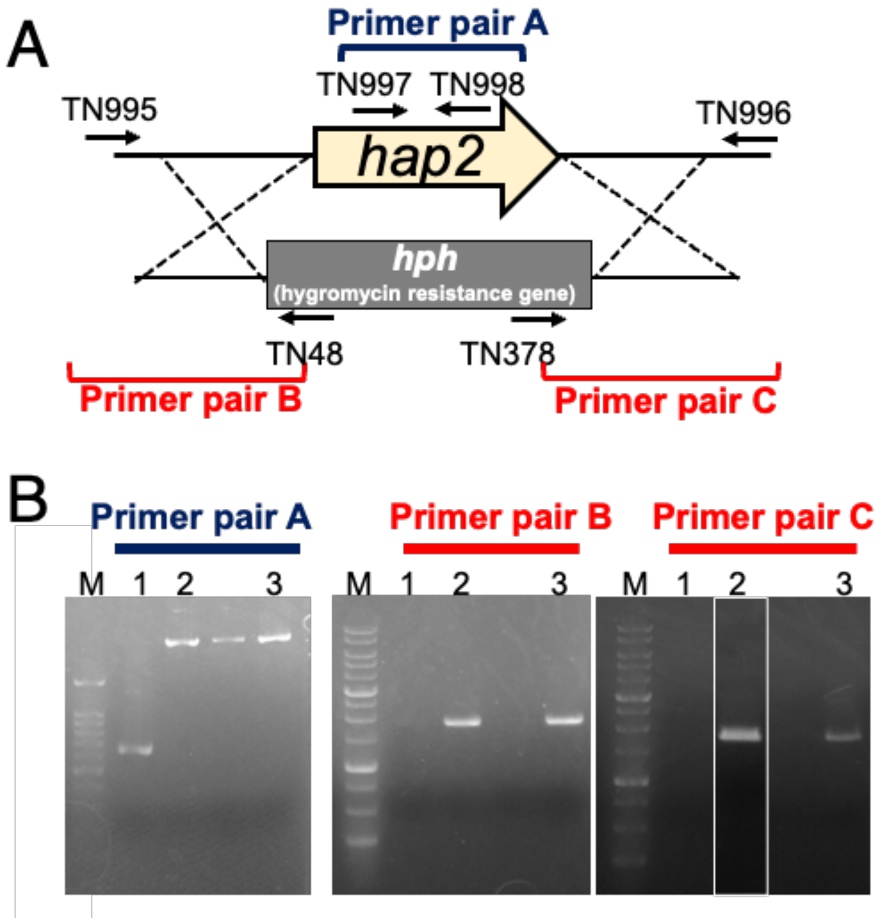


**Fig. S1** Generation of the *hap2* deletants (Δ*hap2*#1 and Δ*hap2*#2) derived from 20b using homologous recombination. (A) A diagram of the genomic locus of *Pleurotus ostreatus hap2*. Black arrows indicate the primers used for the polymerase chain reaction (PCR) amplification. (B) Genomic PCR experiments confirming the *hap2* deletion in the deletants. Primers used in each PCR experiment are shown in (A).

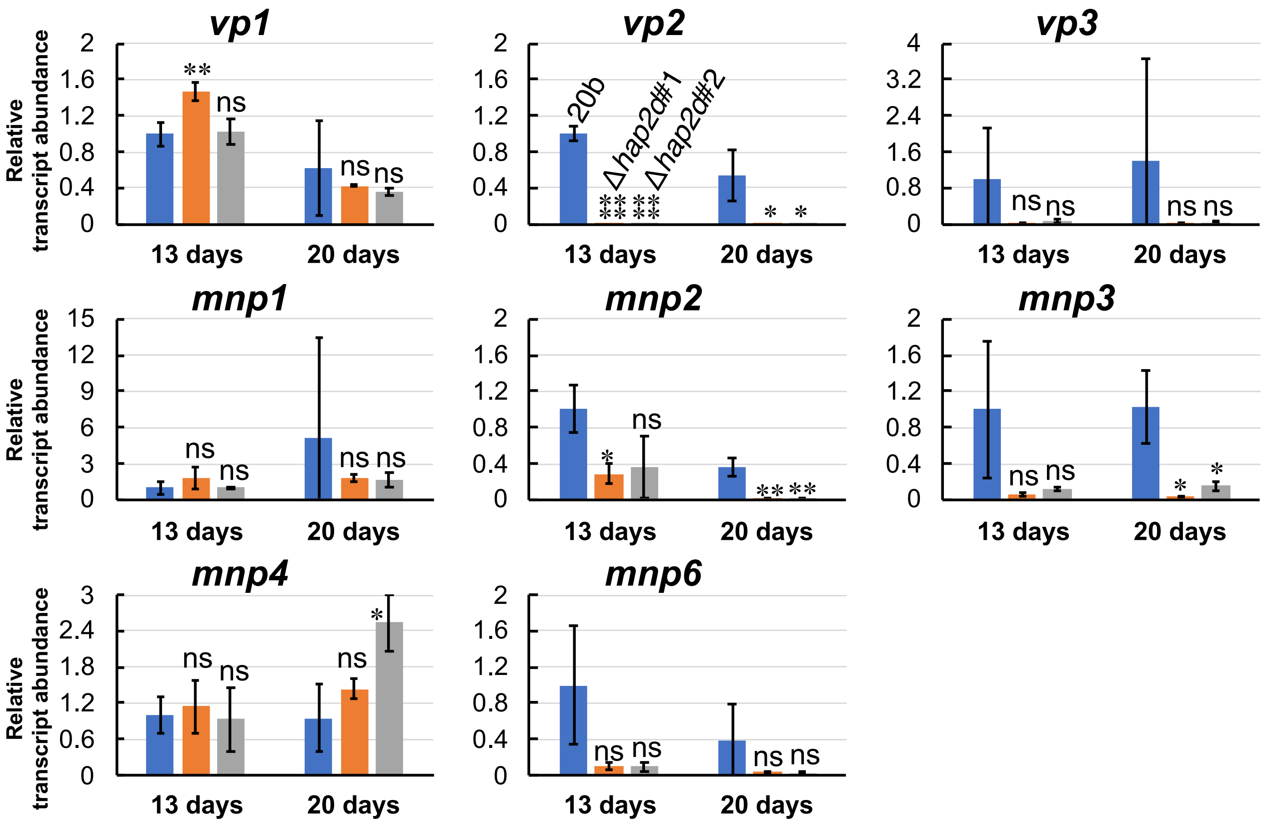


**Fig. S2** Relative transcriptional expression levels of the eight *mnp*/*vp* genes in the *hap2* deletants grown on Holocellulosel-I at 13- and 20-day culture periods (n = 3). Expression levels were standardized by *β-tubulin*, and the expression level of 20b at 13 days is shown as 1. Graphs indicate mean values and bars indicate standard deviations. Statistical significance tests between the indicated two strains at the respective culture periods were performed using a two-tailed equal variance *t*-test. *$p < 0.05$, **$p < 0.01$, ***$p < 0.001$. "ns" indicates statistical non-significance ($p > 0.05$).

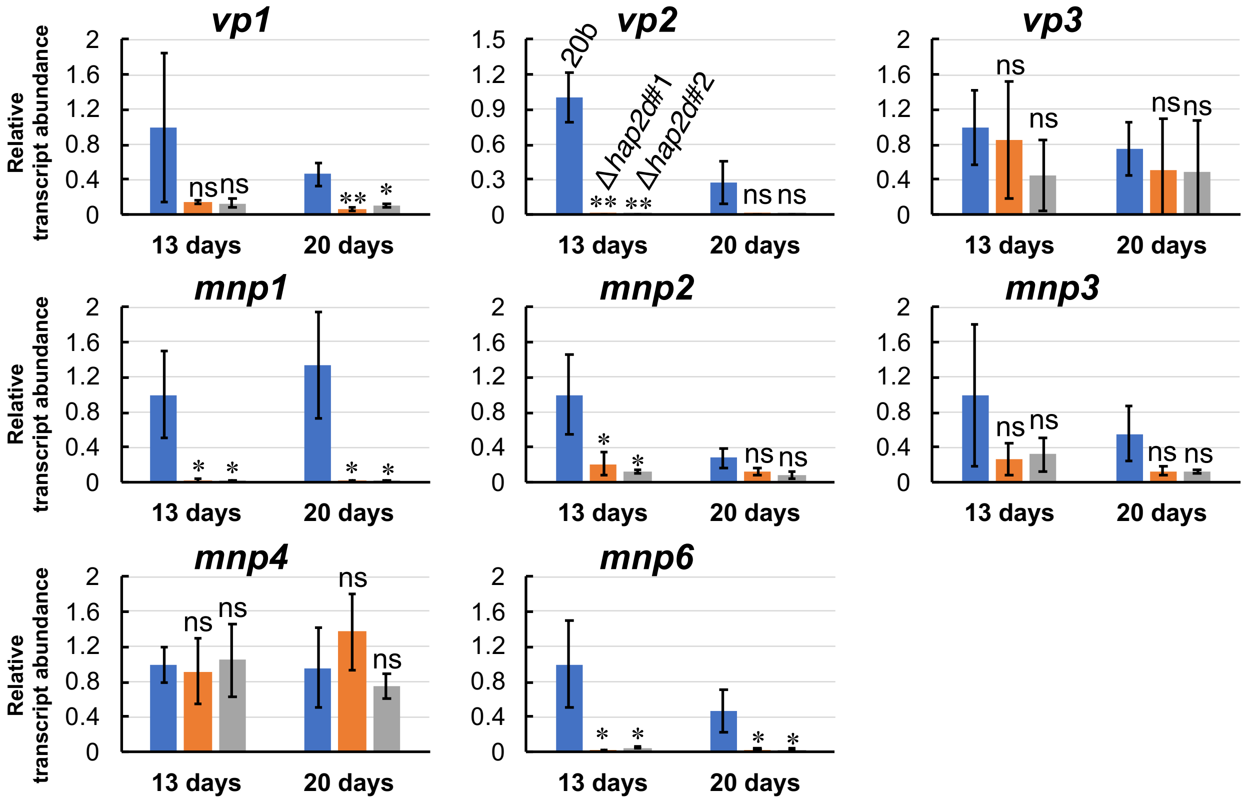


**Fig. S3** Relative transcriptional expression levels of the eight *mnp*/*vp* genes in the *hap2* deletants grown on Avicel-I at 13- and 20-day culture periods (n = 3). Expression levels were standardized by *β-tubulin*, and the expression level of 20b at 13 days is shown as 1. Graphs indicate mean values and bars indicate standard deviations. Statistical significance tests between the indicated two strains at the respective culture periods were performed using a two-tailed equal variance *t*-test. *$p < 0.05$, **$p < 0.01$, ***$p < 0.001$. "ns" indicates statistical non-significance ($p > 0.05$).

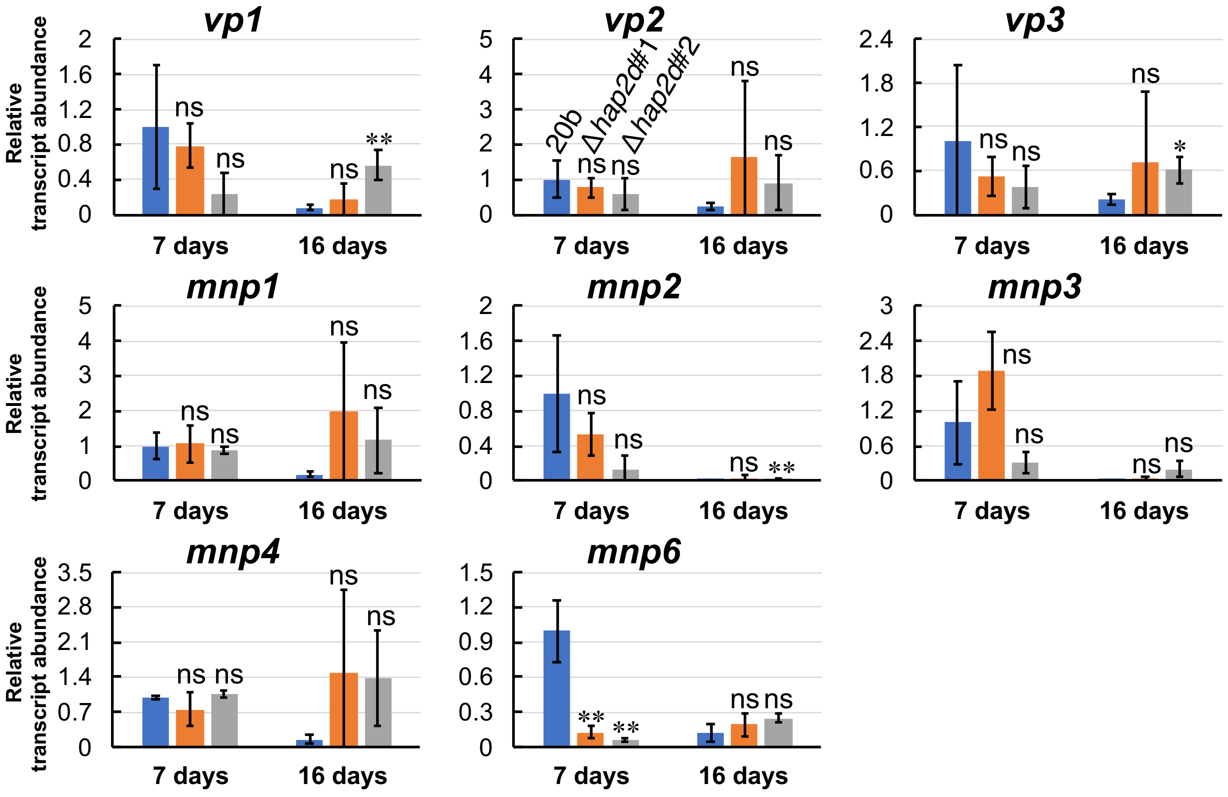


**Fig. S4** Relative transcriptional expression levels of the eight *mnp*/*vp* genes in the *hap2* deletants grown on YMG agar plates at 7- and 16-day culture periods (n = 3). Expression levels were standardized by *β-tubulin*, and the expression level of 20b at 7 days is shown as 1. Graphs indicate mean values and bars indicate standard deviations. Statistical significance tests between the indicated two strains at the respective culture periods were performed using a two-tailed equal variance *t*-test. *$p < 0.05$, **$p < 0.01$, ***$p < 0.001$. "ns" indicates statistical non-significance ($p > 0.05$).
